# Hierarchical Manipulation Skill Learning for Long-Horizon Robotic Manipulation Based on Residual Finite Scalar Quantization

**DOI:** 10.3390/s26196310

**Published:** 2026-10-06

**Authors:** Guanghui Ma, Jiahui Guo, Xinhua Tang, Huaidong Zhou, Yongfeng Rong

**Affiliations:** 1 School of Integrated Circuits, Anhui Polytechnic University, Wuhu 241000, China; maguanghui@ahpu.edu.cn; 2 School of Automation, Northwestern Polytechnical University, Xi’an 710072, China; guojiahui@mail.nwpu.edu.cn; 3 School of Artificial Intelligence, Shenzhen University, Shenzhen 518060, China; 18379498580@163.com; 4 Department of Computer Science and Technology, Tsinghua University, Beijing 100084, China; yfrong@tsinghua.edu.cn

**Keywords:** long-horizon robotic manipulation, skill learning, action discretization, residual finite scalar quantization, Mamba, Transformer, Meta-World

## Abstract

Leveraging discrete sequence prediction for manipulation skill learning has demonstrated immense potential for complex long-horizon tasks. However, guaranteeing precise and robust execution under complex scenarios remains a core challenge for efficient autonomous robotic operation. This demands execution policies capable of generating precise and efficient action sequences based on perceptual feedback. To address the loss of fine-grained action details induced by action discretization and the difficulty in preserving long-horizon temporal dependencies in long-horizon tasks, this work proposed a hierarchical manipulation skill learning method based on residual finite scalar quantization (RFSQ). At the action representation level, a robust RFSQ with an adaptive linear scaling mechanism was introduced. It effectively mitigated residual decay in multi-level action quantization and realized accurate discrete reconstruction of continuous action sequences. At the action sequence generation level, a hybrid hierarchical architecture integrating Mamba2 and Transformer was constructed. Mamba2 efficiently captured temporal dependencies among actions, while Transformer precisely modeled fine-grained residual dependencies across action quantization hierarchies. A multimodal perception fusion module was further incorporated to strengthen instruction-semantic alignment on language-instructed tasks. A series of experiments were conducted on the LIBERO and Meta-World simulation environments. Experimental results demonstrated that the proposed method achieved outstanding performance in multi-task and long-horizon task learning.

## 1. Introduction

With the rapid advancement of artificial intelligence and robotics, enabling robots to adapt to unstructured complex environments and perform long-horizon multi-stage manipulation tasks has become a prominent research topic in the field of embodied intelligence [1,2,3]. To realize this goal, low-level manipulation control capability constitutes one of the essential core capabilities of robotic systems, which can generate precise, coherent, and highly generalized manipulator actions according to environmental feedback. Given that traditional pre-programming methods struggle to handle dynamic unstructured scenarios, constructing a learning-based control policy that translates sparse semantic instructions into low-level actions represents a promising direction [4].

Imitation learning and behavior cloning (BC) provide a practical route for learning manipulation skills from demonstrations. However, direct regression of continuous actions with mean squared error tends to average multimodal demonstrations and may produce unstable or physically invalid actions. Generative policies based on energy functions or diffusion models improve the modeling of multimodal action distributions, yet their iterative inference pipelines introduce non-negligible latency in high-frequency control loops [5,6,7]. Another prominent research direction frames action prediction as a sequence modeling problem, instantiated via techniques such as action chunking or autoregressive token prediction. These approaches are attractive because they can exploit the scalability of modern sequence models, but they also require a reliable discretization of continuous robot actions [8,9,10].

Discrete action representations have been explored through vector quantization (VQ), finite scalar quantization (FSQ), and so on [11,12,13]. Vector-quantized representations can transform continuous trajectories into compact codebook indices, but they may suffer from codebook collapse, under-utilization of entries, or sensitivity to the learned embedding space. FSQ avoids a learned vector codebook by quantizing each latent dimension with predetermined levels, which makes the codebook implicit and easy to utilize. Nevertheless, when residual quantization is directly applied to robotic action chunks, residual magnitudes may decay rapidly with deeper quantization layers. Later quantization layers then operate near dead zones, reducing their ability to preserve fine action details. At the sequence modeling level, Transformer-based policies are effective at representing cross-token dependencies, but they exhibit quadratic computational complexity and susceptibility to long-context degradation become increasingly problematic for long-horizon manipulation [8,9,14]. State-space models (SSMs), particularly Mamba and Mamba2, provide a promising alternative because they can capture long-horizon temporal patterns with linear complexity [15,16,17,18]. However, the residual hierarchy within a quantized action token is not purely temporal; it is a fine-grained dependency across residual code layers. This suggests that temporal modeling and residual-level modeling should be separated rather than handled by a single architecture.

At the level of low-level manipulation skill execution, converting semantic instructions planned at the high level into precise control signals represents another critical technical issue to be addressed. Although end-to-end vision–language–action (VLA) models leverage the strong sequence fitting capability of Transformers to directly output actions, such methods heavily rely on massive embodied cross-domain data and suffer from prohibitive training costs and data distribution bias in specific industrial or service scenarios [19]. More importantly, to adapt to sequence generation models, existing works such as RT-1 mostly adopt coarse-grained discrete action representations, which frequently lead to the loss of high-frequency action details [20]. Meanwhile, for long-contact and multi-stage tasks, the sharp increase in action sequence length makes the model prone to error accumulation and catastrophic forgetting of historical states.

To address the loss of fine-grained action details induced by action discretization and the difficulty in preserving long-horizon temporal dependencies during long-horizon robotic manipulation tasks, this work proposed a hierarchical manipulation skill learning method based on residual finite scalar quantization (HMSL-RFSQ). The main contributions of this work are summarized as follows: (1) It introduces a robust residual finite scalar quantization (RFSQ) equipped with an adaptive linear scaling mechanism to effectively mitigate residual decay in multi-level action quantization. (2) It develops a hybrid hierarchical architecture integrating Mamba2 and Transformer; Mamba2 efficiently captured temporal dependencies among actions, while Transformer precisely modeled fine-grained residual dependencies across action quantization hierarchies. (3) Experiments are conducted on the LIBERO and Meta-World simulation environments; the results indicate that the proposed method achieves substantial improvements over the selected baselines [21,22].

The remainder of this paper is organized as follows: Section 2 reviews related work; Section 3 contains the description of the proposed method; comparative experiments, ablation studies and visualization analyses are performed to verify the proposed method, and the experimental results are presented and discussed in Section 4; Section 5 concludes this work and outlines potential directions for future research.

## 2. Related Work

Robot manipulation skill learning aims to construct nonlinear mapping policies from high-dimensional perceptual inputs (e.g., vision, language instructions, etc.) to low-level motion control, empowering robots with the capability to execute complex tasks in unstructured environments. Currently, research in this field mainly focuses on three mainstream directions: first, leveraging generative models to tackle the multimodal distribution problem of action data; second, adopting architectures such as Transformer to reformulate action generation as a sequence prediction task; and third, improving the sample efficiency of policy learning by constructing compact latent-space representations.

### 2.1. Manipulation Skill Learning Based on Generative Policies

Complex tasks inherently exhibit multimodality: given an identical visual observation, experts often have multiple valid manipulation trajectories to complete the task. Conventional behavior cloning methods generally adopt the mean squared error (MSE) loss function, which is mathematically equivalent to averaging all demonstration trajectories. Such modal averaging yields intermediate states that violate physical constraints, resulting in jitter or stagnation of the robotic manipulator’s motions. To address this challenge, generative models have gradually emerged as one of the core paradigms in this field.

Florence et al. proposed Implicit Behavioral Cloning (IBC), which introduced an inference framework based on energy models [5]. The method trained an energy function E(s,a) to measure the matching degree between observations and actions and searched for the action with the minimum energy via sampling optimization during inference. Although IBC demonstrated the expressive power of generative models for non-convex distributions, it incurred heavy computational costs in iterative optimization and tended to fall into local minima, so it failed to meet high-frequency control requirements. To balance fitting capacity and inference efficiency, Diffusion Policy modeled action prediction as a conditional denoising process [6]. The model took visual observations as conditions and utilized the network to perform multi-step iterative denoising on Gaussian noise, directly outputting action sequences that conformed to expert distributions. It preserved multimodal characteristics while guaranteeing trajectory smoothness. Furthermore, Pearce et al. optimized diffusion-based behavioral cloning to tackle the drawback that conventional methods struggled to model correlations across action dimensions [7]. The study adopted a lightweight architecture and disabled classifier-free guidance to avoid bias toward low-probability actions. Meanwhile, the Diffusion-X and Diffusion-KDE sampling strategies proposed enabled accurate modeling of conditional action distributions p(a∣o) and effectively mitigated the risk of distribution shift in offline learning.

For three-dimensional (3D) spatial manipulation, 3D Diffuser Actor took 3D point clouds as inputs and learned SE(3)-equivariant features, which endowed the diffusion policy with strong robustness against variations in object poses [23]. To address the challenge of cross-environment adaptation, AdaptDiff introduced a guidance-based fine-tuning mechanism [24]. It iteratively optimized actions during the generation phase, leveraging reinforcement learning feedback to flexibly adapt to obstacles and dynamic constraints in new environments. To tackle the limitations of slow inference and inflexible temporal patterns in traditional diffusion models, recent studies optimized their generation paradigms. Consistency Policy introduced consistency distillation to predict trajectory endpoints and compressed sampling into merely one or two steps, which drastically cut down inference latency [25]. Diffusion Forcing combined next-token prediction with full-sequence diffusion [26]. It realized flexible generation of variable-length sequences while retaining the advantages of distribution fitting. In addition, the π0 model built upon a mixture-of-experts architecture and integrated flow matching techniques into joint vision–language–action modeling [27]. The method combined optimal transport paths with ordinary differential equation (ODE) solvers, balancing the precision and real-time performance of continuous action generation, and effectively supported end-to-end complex dexterous manipulation.

### 2.2. Manipulation Skill Learning Based on Sequence Modeling

Robotic manipulation is inherently a continuous spatiotemporal decision-making process. Overcoming compounding errors from single-step prediction and capturing long-horizon temporal dependencies constitute the key to improving skill robustness. For this reason, modeling action execution as sequence prediction via architectures such as Transformer becomes the mainstream paradigm at present. ACT proposed an action chunking mechanism [8]. Its decoder predicted complete action sequences within a future time window conditioned on current observations. By leveraging temporal-integration techniques, the method precisely captured temporal coherence of actions and effectively eliminated compounding errors, thereby enabling the learning of high-precision manipulation skills. Subsequently, Mobile ALOHA extended this sequence modeling mechanism to full-body coordinated control and further verified its effectiveness when processing high-dimensional proprioceptive sensing data [28].

In the field of offline reinforcement learning, Q-Transformer discretized the Q-value function in segments and adopted Transformer to perform autoregressive prediction [9]. The approach realized long-horizon credit assignment via full-sequence attention mechanisms, which overcame the limitations of single-step reward backpropagation in conventional Bellman equations. As a result, it accurately identified critical actions within suboptimal datasets filled with numerous invalid attempts and optimized the policy accordingly. In terms of spatial information serialization, PerAct reconstructed RGB-D observations into 3D voxel patches, fused them with language instructions, and flattened the combined features into sequential inputs [10]. Instead of predicting continuous actions, the method formulated manipulation as a classification task that predicted the next optimal discrete keyframe. The key poses output by the model were eventually passed to low-level motion planners for execution. In addition, Octo proposed a universal Transformer policy interface [29]. The architecture unified the encoding of multimodal data through modality-specific tokenizers and successfully adapted to heterogeneous observation and action spaces of cross-embodiment robots. With pretraining conducted on massive cross-dataset trajectories, this work fully validated the potential of large-scale sequence modeling for universal skill learning.

In terms of high-level semantic sequence learning, the “Next-Token Prediction” paradigm of vision–language models (VLMs) was introduced into low-level robot control. RT-2 discretized the robot’s 6-DoF (six degrees of freedom) poses and gripper actions into tokens and directly converted multimodal inputs into sequence generation tasks [30]. To address the precision loss caused by action discretization, OpenVLA integrated visual features and a language backbone to construct a unified vision–language–action (VLA) architecture [31]. The method directly mapped actions to language tokens for predictive optimization and effectively bridged the discrepancy between semantic representations and continuous skill expressions. The recently proposed RDT adopted the Diffusion Transformer as a scalable backbone to replace conventional diffusion architectures [32]. By unifying multi-robot action spaces and optimizing multimodal encoding, the model not only successfully processed long-sequence data contaminated by physical noise but also exhibited outstanding cross-task generalization and zero-shot transfer capabilities.

### 2.3. Manipulation Skill Learning Based on Latent-Space Representations

The joint space of robotic manipulators contained redundancies and lacked structured priors, so direct end-to-end learning frequently suffered from difficulties in action decoupling and poor sample efficiency. Accordingly, constructing a low-dimensional, compact skill latent space with favorable structural properties emerged as a critical solution. The SPiRL framework leveraged deep latent variable models to extract skill priors from large-scale unstructured datasets [33]. The approach compressed action sequences into low-dimensional continuous latent variables. The high-level policy only needed to output low-frequency signals, and the decoder reconstructed smooth trajectories from these signals, which drastically reduced the dimensionality of the action search space.

To address the mode ambiguity issue that frequently arose within continuous latent spaces, VQ-BeT integrated VQ techniques to learn discrete skill codebooks [34]. Such discretized representations clearly demarcated boundaries between distinct skills and markedly boosted the consistency of action generation when the model processed long-horizon tasks. In terms of integrating visual representations with learned skills, R3M leveraged large-scale human egocentric videos to construct temporally and semantically fused latent spaces [35]. Through temporal contrastive learning and video–language alignment, the method transformed intricate visual information into low-dimensional embeddings that matched robotic skills well. To further deepen the effects of language supervision, Voltron introduced collaborative optimization under dual objectives of visual reconstruction and language generation based on a masked autoencoding (MAE) backbone [36]. This framework constructed a unified latent-space representation for vision and language. It accurately responded to high-level semantic instructions while encoding low-level spatial features and effectively remedied the drawback that conventional visual representations remained insensitive to linguistic inputs.

To realize deep alignment between language instructions and low-level skills, LISA proposed a hierarchical imitation learning paradigm [37]. By constructing a skill abstraction layer, the method mapped language and observations into discrete skill codes to guide low-level action generation. On this basis, SkillDiffuser designed a hybrid architecture integrating skill prediction and vector quantization [38]. Guided by the discrete skill latent space, the model adopted a low-level diffusion model to generate trajectories consistent with semantic instructions. In addition, for complex long-horizon planning tasks, MimicPlay learned a hierarchical latent planner from low-cost human demonstrations [39]. Instead of directly predicting actions, this model predicted future target states within the latent space to steer the low-level policy, which drastically improved the execution success rate of long-horizon tasks.

In summary, although the studies have achieved remarkable progress in various dimensions, they still encounter substantial challenges when tackling complex long-horizon manipulation tasks. The inherent iterative denoising mechanism of generative policies brings considerable inference latency, which fails to satisfy the real-time requirements of robotic manipulation. The lossy compression property of discrete latent-space representations inevitably leads to the loss of fine-grained action details and degrades the precision of action execution. Meanwhile, Transformer-based autoregressive architectures suffer from drastically soaring computational overhead when tackling long-horizon tasks. Furthermore, they encounter the decay of long-horizon contextual memory, which causes deviations from task expectations and eventually results in task failure.

## 3. Method

### 3.1. Overview of HMSL-RFSQ

**Figure 1:**
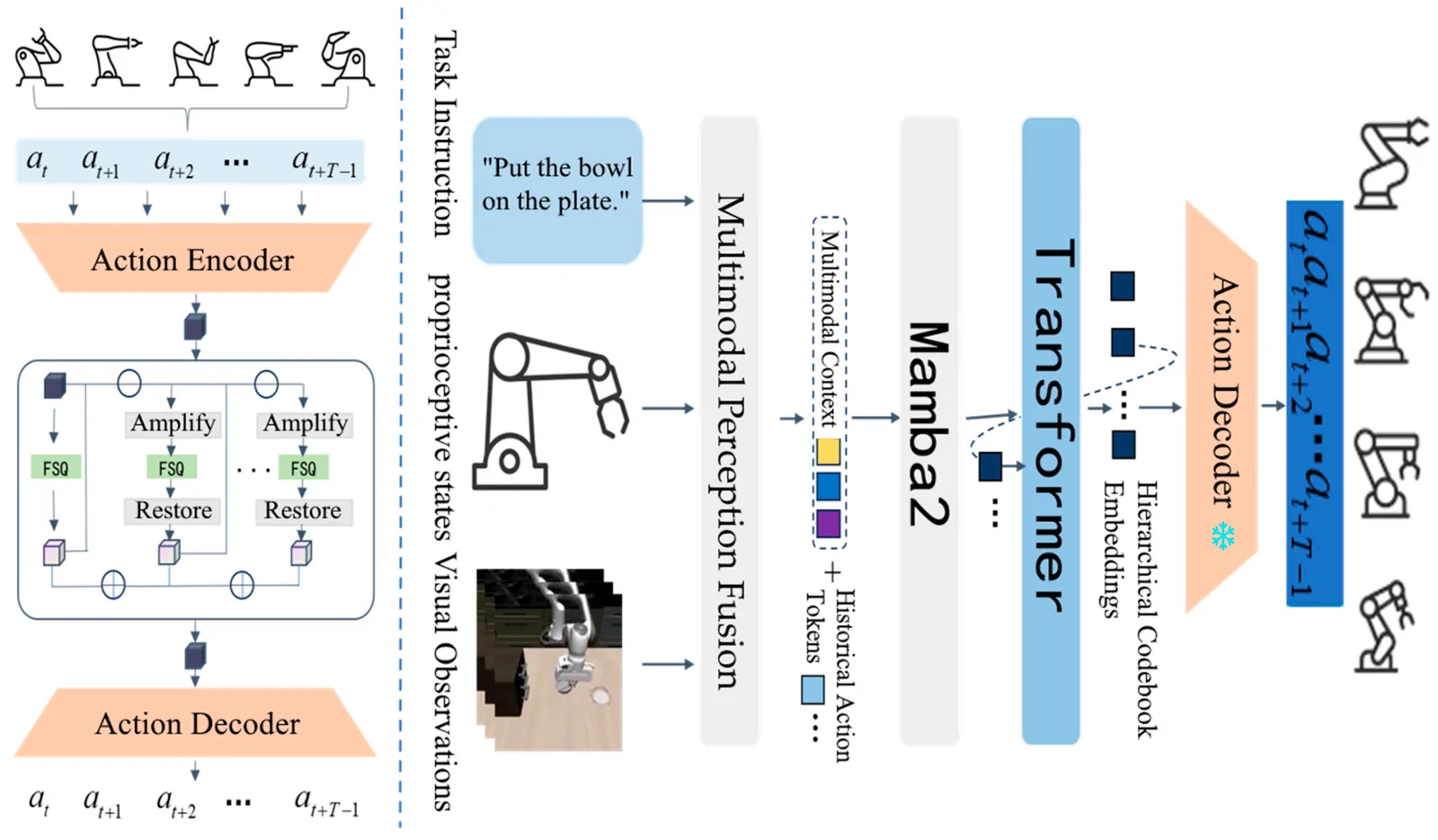
Architecture of HMSL-RFSQ.

This work proposed HMSL-RFSQ, a robotic manipulation model framework built on RFSQ and a hybrid Mamba-Transformer architecture, as illustrated in Figure 1. The overall model consists of two core modules, discrete action representation and skill generation, and its operating workflow covers three sequential stages. In the first stage (action discretization, left side of Figure 1), the high-dimensional continuous action sequence $A_{t}$ is compressed into temporal features via a causal convolutional encoder and is then mapped into multi-layer discrete indices $Z_{t}$ through the robust RFSQ with high reconstruction fidelity. This procedure constructs a high-precision discrete codebook and lays a solid foundation for subsequent policy learning. In the second stage (skill generation, right side of Figure 1), targeting the two-dimensional temporal-hierarchical property of discrete action sequences, this work designs a hybrid hierarchical policy network based on Mamba-Transformer. Conditioned on current multimodal observations $o_{t}$ and task instructions l, this architecture leverages Mamba2 to capture long-horizon dependencies along the temporal dimension and adopts Transformer decoder to process residual dependencies along the hierarchical dimension, thereby autoregressively predicting future action indices. During the deployment and inference stage, the system forms a closed perception–decision–execution loop. The policy samples prospective skill indices ${\hat{Z}}_{t}$ according to real-time environmental observations, and the decoder from the first stage reconstructs continuous actions ${\hat{A}}_{t}$ from these indices.

### 3.2. Problem Definition

Given a limited dataset of expert demonstrations D={O,L,A} robotic manipulation skill learning is formulated as a multimodal conditional sequence prediction and generation task, whose input and output spaces are described by the tuple M=(O,A,L,H).

Observation Space O: It contains partial observations received by the agent at timestep t, denoted as $o_{t}\in O$. In this work, $o_{t}$ includes high-dimensional RGB image inputs $I_{t}$ and proprioceptive robot sensing information (e.g., joint angles, end-effector poses, gripper states) $p_{t}$.

Action Space A: It is defined as the continuous space of robotic joint position or velocity commands, namely $a_{t}\in {\mathbb{R}}^{d}$.

Language Instruction Space L: l∈L represents unstructured natural language task instructions (e.g., “Open the drawer and place the black bowl inside the drawer”), which guided the robot’s manipulation intentions.

Prediction Horizon H: To capture long-horizon temporal dependencies and guarantee action coherence, the model does not predict single-step actions. Instead, it predicts an action sequence spanning a future temporal chunk at timestep t, denoted as $A_{t}=\{a_{t},a_{t+1},\ldots ,a_{t+H-1}\}$.

Considering that the mapping directly from observations to continuous action sequences $f:(O,L)\to A^{H}$ is highly susceptible to multimodal distributions and thereby suffers from mean drift, this work introduces a discrete latent skill space Z as an intermediate representation. Accordingly, the original problem is decomposed into two coupled subproblems: action representation learning and prior policy learning. The action representation learning module learns an encoding–decoding mapping that projects continuous action sequences $A_{t}$ onto the discrete space Z, namely $A_{t}↔Z_{t}$, where $Z_{t}\in {\mathbb{Z}}^{H\times K}$; K denotes the number of residual layers for discrete encoding. At the prior policy learning stage, the model needs to learn the conditional probability distribution $p(Z_{t}|o_{t},l)$ of discrete skill indices $Z_{t}$ given observations $o_{t}$ and task instructions l. Consequently, the overall optimization objective of the model is transformed into maximizing the log-likelihood over the expert demonstration dataset D.

(1) $$\max_{\theta ,\psi }E_{(o,l,a)\sim D}\left[\log p_{\psi }(A_{t}|Z_{t})+\log p_{\theta }(Z_{t}|o_{t},l)\right],$$

where ψ stands for the action decoder, and θ represents the parameters of the prior policy model. The first term $\log p_{\psi }(A_{t}|Z_{t})$ guarantees that the discrete codebook reconstructed continuous actions with low reconstruction error, while the second term $\log p_{\theta }(Z_{t}|o_{t},l)$ ensures that the policy accurately predicts discrete sequences conditioned on perceptual inputs.

### 3.3. Action Representation Network Based on Robust RFSQ

This module aimed to construct an accurate encoding–decoding mapping channel between the continuous action space A and the discrete skill space Z. To address layer-wise signal attenuation and codebook collapse in conventional discretization methods, an action representation network equipped with robust RFSQ was designed. The model consisted of an action encoder–decoder and a residual quantization enhanced by adaptive linear scaling, and it was optimized end-to-end via self-supervised learning objectives.

#### 3.3.1. Action Encoder

**Figure 2:**
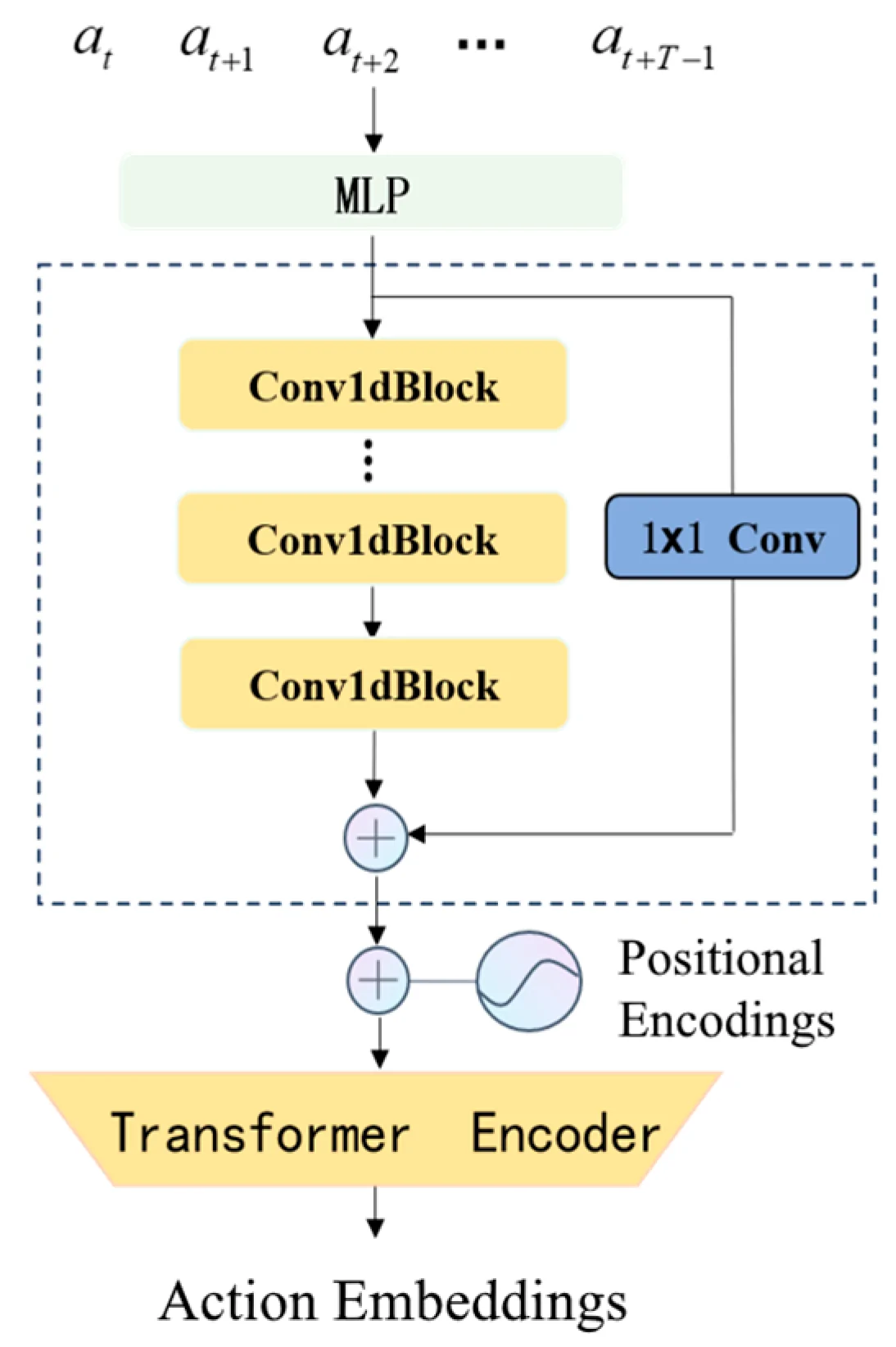
Action encoder.

The action encoder ϕ adopted a hierarchical asymmetric structure to build the mapping from the raw action space to the latent representation space. This component was formed by sequentially stacking linear projection layers, residual temporal convolution blocks and a Transformer encoder, as depicted in Figure 2.

The input action sequences $A_{t}\in {\mathbb{R}}^{T\times d}$ (T>H) were first mapped to the model dimension $d_{m}$ via a linear projection layer and then fed into residual temporal convolution blocks. This module consisted of N stacked causal convolution units. To strictly satisfy temporal causality of physical systems, asymmetric causal padding was applied to all convolutional layers, which guaranteed that the convolutional output at timestep t only relied on inputs from the current and historical timesteps. The computation process of the i-th convolutional unit was formally defined as follows:

(2) $$h_{\tau }^{\left(i\right)}=h_{\tau }^{\left(i-1\right)}+F_{\mathrm{Mish}}\left(\mathrm{GN}(W^{\left(i\right)}*\mathrm{Pad}(h_{\tau }^{\left(i-1\right)})+b^{\left(i\right)})\right),$$

where $h_{\tau }^{\left(i\right)}$ denotes the hidden state of the i-th layer at timestep t; $W^{\left(i\right)}$ and $b^{\left(i\right)}$ represent the convolutional parameters of the i-th layer; $F_{\mathrm{Mish}}(x)=x\cdot \tanh(\ln(1+e^{x}))$ stands for the nonlinear activation function; Pad(·) refers to the operation of zero-padding on the left side along the temporal axis; and GN(·) means group normalization. To retain critical dynamic features while eliminating high-frequency redundant information, temporal downsampling is performed on the sequence from original length T to H (H=T/F) by setting convolutional strides, where F denotes the downsampling factor.

The compressed feature sequence was denoted as $Z_{conv}\in {\mathbb{R}}^{H\times d_{m}}$. After adding sinusoidal positional encoding $P_{pos}$, the result $Z_{in}=Z_{conv}+P_{pos}$ was fed into the Transformer encoder, which finally output action embeddings $E_{t}\in {\mathbb{R}}^{H\times d}$ enriched with global context for subsequent discrete quantization.

#### 3.3.2. Robust RFSQ with Adaptive Linear Scaling

After processing by the action encoder, continuous action sequences were mapped to action embeddings $E_{t}\in {\mathbb{R}}^{H\times d}$. To achieve efficient autoregressive generation, the continuous embeddings $E_{t}$ had to be converted into discrete tokens. When conventional vector quantization was adopted for action quantization, it suffered from severe precision degradation, which frequently required an extra continuous regression head for compensation and introduced uncertainties in both training and inference. Although finite scalar quantization outperformed traditional vector quantization in stability thanks to its property of eliminating explicit codebook maintenance, single-layer FSQ imposed a hard limit on the capacity of its implicit codebook due to predefined scalar dimensions and quantization levels. Such coarse-grained discretization failed to reconstruct intricate fine-grained action details accurately.

To achieve discretization with both compactness and strong expressive power, this work adopted an RFSQ strategy. Specifically, $E_{t}$ was regarded as a set composed of H single-step feature vectors, namely $E_{t}=\{e_{1},e_{2},\ldots ,e_{H}\}$; for each feature vector $e_{h}$, cascaded quantizers decomposed the feature into superposed residuals ranging from coarse to fine granularity. Nevertheless, directly applying FSQ to multi-layer residual structures brought about a severe distribution mismatch between fixed codebooks and dynamic residuals. Concretely, FSQ adopted tanh-based fixed scalar grids as its implicit codebook with a fixed effective dynamic range (normally [−1, 1]). Within the cascaded quantization architecture, the amplitude of residual signals $r_{k-1}$ decayed exponentially as the layer index k increased ($∥r_{k}∥\ll ∥r_{k-1}∥$). When deep-layer residuals became extremely faint, all signals fell entirely into the linear dead zone near the origin of the tanh function, which forced all quantization outputs to zero.

To tackle the problem of residual decay, this work drew on cutting-edge advances in the field of image compression and introduced an adaptive linear scaling mechanism. Precise capture of residuals from each layer was realized via a closed-loop pipeline of “amplification–quantization–restoration”. For the first layer of residual quantization, the system directly quantized the original feature $e_{h}\in E_{t}$ without scaling operations:

(3) $$z_{h,1}={\mathrm{FSQ}}_{1}(e_{h}),$$

The residual $r_{h,1}$ after quantization of the first layer was calculated and taken as the input baseline for subsequent quantization layers:

(4) $$r_{h,1}=e_{h}-z_{h,1},$$

Starting from the second quantization layer, the adaptive linear scaling mechanism was adopted to avoid the quantizer falling into dead zones caused by residual decay. For each layer k∈{2,…,K}, the algorithm constructed closed-loop data flow of “amplification–quantization–restoration” to accurately capture faint subtle details. First, a layer-specific learnable scaling factor $\alpha_{k}\in {\mathbb{R}}^{d}$ (exponentially parameterized $\alpha_{k}=\exp(\theta_{k})$ to guarantee positivity) linearly amplified the weak residual $r_{h,k-1}$ from the previous layer, so that the residual fits the codebook boundaries of FSQ:

(5) $${\tilde{r}}_{h,k-1}=\alpha_{k}\cdot r_{h,k-1},$$

The amplified signal was quantized to obtain the discrete quantized codeword $z_{h,k}$ of the current layer:

(6) $$z_{h,k}={\mathrm{FSQ}}_{k}({\tilde{r}}_{h,k-1}),$$

To guarantee physical rigor in residual computation, the quantized codeword $z_{h,k}$ was divided by the scaling factor for restoration before residual updating:

(7) $$r_{h,k}=r_{h,k-1}-z_{h,k}/\alpha_{k},$$

Ultimately, each encoded action feature $e_{h}$ was mapped to K discrete codebook indices $\left\{z_{h,1},\ldots ,z_{h,K}\right\}$. Recursive residual quantization was applied in this process to extract residual information layer by layer. Each layer amplified the quantization error of the preceding layer through adaptive linear transformation so as to precisely capture tiny action details. This progressive bias compensation effectively reduced quantization loss and ensured the improvement of the reconstruction fidelity of discrete representations of continuous actions.

#### 3.3.3. Action Decoder

**Figure 3:**
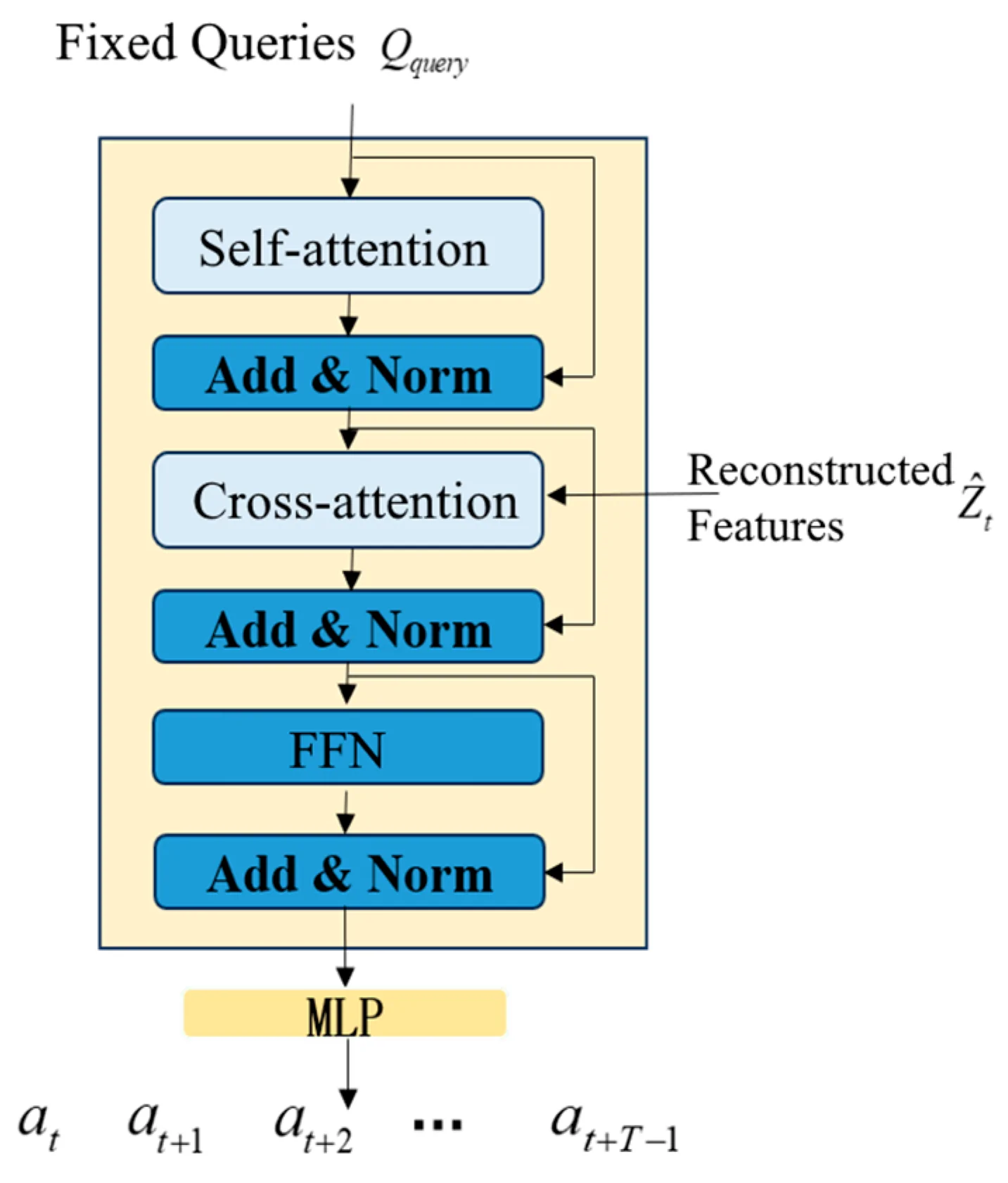
Action decoder.

The decoder ψ aimed to reconstruct continuous action sequences from discrete latent skill indices $Z_{t}\in {\mathbb{Z}}^{H\times K}$. Built upon the Transformer decoder architecture, this module integrated cascaded codebook superposition of RFSQ and a fixed query generation mechanism, as illustrated in Figure 3.

First, for the action index at each timestep, the model linearly superimposed codebook embeddings of all K quantization layers to obtain reconstructed features corresponding to that timestep. Accordingly, skill indices of H action steps were converted into corresponding reconstructed features ${\hat{Z}}_{t}\in {\mathbb{Z}}^{H}$. The reconstructed feature ${\hat{Z}}_{h}$ of the action at each timestep h within the latent space was formally defined as follows:

(8) $${\hat{z}}_{h}=W_{proj}\left(e^{\left(1\right)}(i_{h,1})+\sum_{k=2}^{K}\frac{e^{\left(k\right)}(i_{h,k})}{\alpha_{k}}\right),$$

where $i_{h,k}\in \{1,\ldots ,V\}$ denotes the discrete index of the h-th timestep action at the k-th quantization layer (V represents the codebook size); $e^{\left(k\right)}(\cdot )$ stands for the codebook lookup operation of the k-th layer; and $W_{proj}\in {\mathbb{R}}^{d_{m}\times d_{c}}$ is a learnable projection matrix that mapped superimposed encoded features back to the model dimension, where $d_{m}$ and $d_{c}$ refer to the model dimension and the dimension of finite scalar quantization, respectively.

Notably, the codebook lookup $E_{k}(\cdot )$ already incorporates the inverse de-scaling defined in Equation (7): for k≥2, $E_{k}(z_{k})=FSQ^{-1}(z_{k})/\alpha_{k}$, while for the first layer (k=1), no scaling is applied ($\alpha_{1}\equiv 1$) and $E_{1}(z_{1})=FSQ^{-1}(z_{1})$. This ensures that the decoder reconstruction and the residual-update rule in Equation (7) operate on the identical de-scaled contribution, removing any ambiguity between stored indices and scaled residuals.

To expand the static feature ${\hat{Z}}_{t}$ into dynamic action sequences of length H, this method constructed a set of fixed query sequences $Q_{query}\in {\mathbb{R}}^{H\times d_{m}}$. The sequences were initialized as the sum of zero tensor and fixed sinusoidal positional encoding $P_{fixed}$, namely $Q_{query}=0+P_{fixed}$. The decoding procedure followed standard Transformer decoding logic and finally output the reconstructed continuous action sequences ${\hat{A}}_{t}$.

### 3.4. Hierarchical Skill Generation Network Based on Mamba-Transformer

After continuous robotic action sequences were mapped into the codebook via discrete quantization, the task in the second stage was transformed into a conditional sequence generation problem: the model autoregressively predicted future skill sequences conditioned on current multimodal observations $o_{t}$ and task instructions l. In the field of robotic manipulation, on the one hand, long-horizon task planning demanded action sequences spanning dozens to hundreds of timesteps, which required models to deliver ultra-low long-sequence inference latency and GPU memory footprint. On the other hand, fine-grained manipulation tasks forced the model to align pixel-level visual features and perform high-precision action refinement. These two requirements imposed dual temporal and precision challenges on generative models. To tackle such issues, this work designed a hybrid generative architecture integrating Mamba2 and Transformer.

#### 3.4.1. Multimodal Perception Fusion

**Figure 4:**
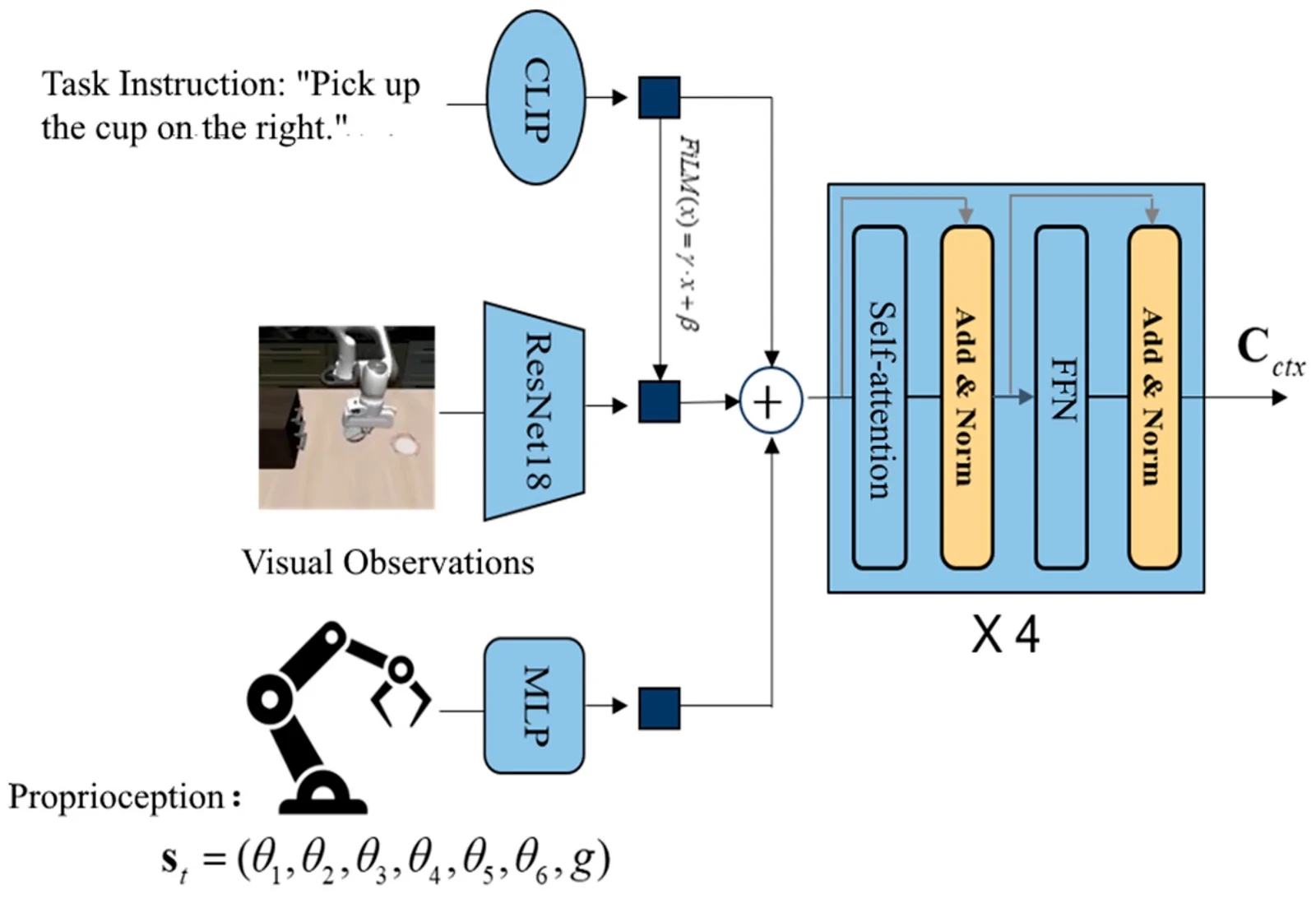
The module of multimodal perception fusion.

Constructing deep correlations between perceptual inputs and task objectives was critical for precise robotic manipulation. In view of the inherent heterogeneity of visual and linguistic features, conventional simple concatenation failed to achieve effective cross-modal alignment. Accordingly, this work built a multimodal perception fusion module, as shown in Figure 4. Leveraging dual mechanisms of spatial-level modulation and semantic-level interaction, the module deeply fused heterogeneous features to produce context information abundant in task semantics.

First, to endow visual features with task-aware capability, the visual encoder adopted ResNet-18 embedded with the Feature-wise Linear Modulation (FiLM) mechanism, while a pre-trained CLIP (Contrastive Language–Image Pretraining) encoder was utilized for language feature extraction. Let $e_{lang}\in {\mathbb{R}}^{D_{L}}$ denote the language embeddings extracted by CLIP and $F_{vis}\in {\mathbb{R}}^{C\times H\times W}$ represent the visual feature map of a certain layer. The FiLM generator computed channel-wise scaling coefficients $\gamma =f_{\gamma }(e_{lang})$ and shift coefficients $\beta =f_{\beta }(e_{lang})$ via two separate linear projection layers, $f_{r}$ and $f_{\beta }$. Affine transformation was then performed on each channel c of the feature map:

(9) $$\mathrm{FiLM}(F_{vis}^{\left(c\right)}|e_{lang})=\gamma_{c}\cdot F_{vis}^{\left(c\right)}+\beta_{c},$$

This mechanism effectively amplified the response of visual features relevant to task instructions (such as the color and texture of target objects) and suppressed background noise. To realize multimodal semantic alignment, FiLM-modulated and flattened visual features, language embeddings, and projected proprioceptive states were concatenated along the sequence dimension to form joint input sequences. After the sequences were processed by four self-attention layers, the output features effectively integrated scene geometric structures and task semantic guidance features $C_{ctx}$, providing a reliable retrieval source for subsequent action sequence generation.

#### 3.4.2. Hierarchical Skill Generation Architecture Based on Mamba-Transformer

**Figure 5:**
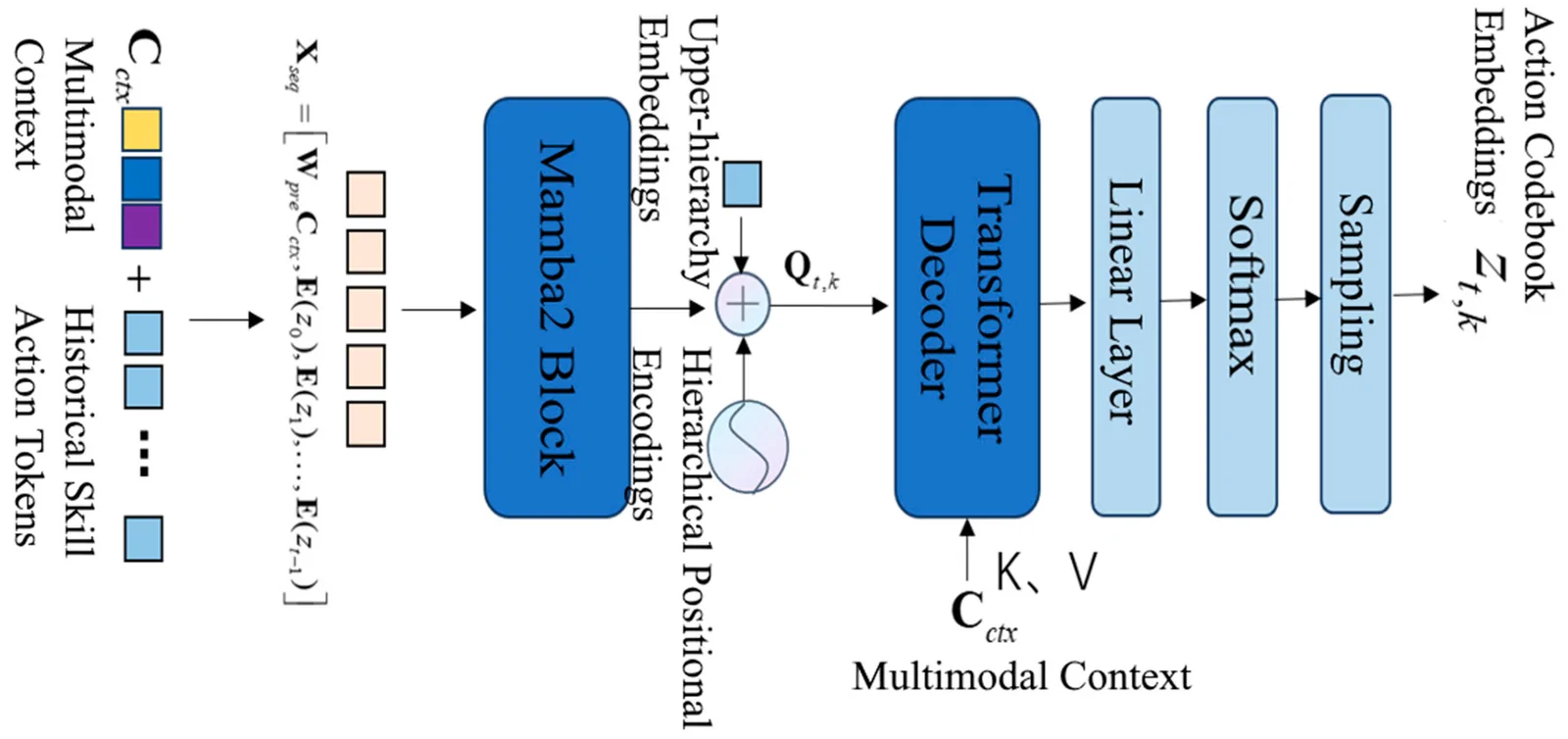
Hierarchical skill generation architecture based on Mamba-Transformer.

To accommodate the distinct dependency characteristics of skill tokens along the temporal dimension H and hierarchical dimension K, this work designed a hybrid generative architecture, as illustrated in Figure 5.

Action sequences carried extremely long-horizon dependencies along the temporal axis H. To efficiently infer future motion trends from historical trajectories, this architecture adopted the Mamba2 block as its temporal backbone. The deeply fused multimodal feature $C_{ctx}$ obtained beforehand was treated as the prior condition for temporal reasoning. Before feeding data into Mamba, $C_{ctx}$ was first mapped to the hidden layer dimension via linear projection and prepended to the front of the historical skill token sequence, forming a joint input sequence:

(10) $$X_{seq}=\left[W_{pre}C_{ctx},E(z_{0}),E(z_{1}),\ldots ,E(z_{t-1})\right],$$

The physical implication of this design lay in the fact that the internal hidden states of Mamba had already been initialized with environmental information before Mamba processed any historical actions. This allowed Mamba to interpret the semantics of historical trajectories based on the current environmental states.

The core of Mamba lay in the selective state-space model. By introducing input-dependent parameters, it discretized the dynamical equations of continuous-time systems. Given the input vector $x_{t}$ (derived from $X_{seq}$) at timestep t, the model first generated the timestep parameter $\Delta_{t}$, input control matrix $B_{t}$, and output control matrix $C_{t}$ via linear projections. It then calculated the discretized state transition matrix ${\bar{A}}_{t}$ and input matrix ${\bar{B}}_{t}$ following the zero-order hold (ZOH) principle:

(11) $$\Delta_{t}=\mathrm{Softplus}(x_{t}W_{\Delta }),\;B_{t}=x_{t}W_{B},\;C_{t}=x_{t}W_{C},$$

(12) $${\bar{A}}_{t}=\exp(\Delta_{t}A),\;{\bar{B}}_{t}={\left(\Delta_{t}A\right)}^{-1}(\exp(\Delta_{t}A)-I)\cdot (\Delta_{t}B_{t}),$$

Recursive state updates and output computations were executed in the final step:

(13) $$h_{t}={\bar{A}}_{t}h_{t-1}+{\bar{B}}_{t}x_{t},$$

(14) $$G_{t}=(C_{t}h_{t})⊙\sigma (x_{t}W_{gate}),$$

where $h_{t}\in {\mathbb{R}}^{D\times N}$ denotes the compressed memory of manipulation history, D represents the feature dimension, and N stands for the dimension of the state space. $G_{t}\in {\mathbb{R}}^{D}$ refers to the multimodal temporal feature integrated with environmental perception, which was obtained through inference.

After obtaining the multimodal temporal feature $G_{t}$, the system parsed it into K coarse-to-fine hierarchical skill indices corresponding to the same timestep. Considering the inherent advantages of the standard Transformer decoder in processing strongly correlated sequences and multimodal queries, this work designed a temporal action context-driven skill generator by introducing input query adaptation and visual feature realignment mechanisms on top of the Transformer backbone.

Instead of simply treating the multimodal temporal feature $G_{t}$ as a raw input, the study regarded it as a global query constraint, namely input query adaptation. Specifically, $G_{t}$ underwent linear projection $W_{proj}$ and was additively injected into the input query of the Transformer decoder:

(15) $$Q_{t,k}=E(z_{t,k-1})+P_{level}(k)+W_{proj}(G_{t}),$$

where $E(z_{t,k-1})$ denotes the embedding of the previous action hierarchy, and $P_{level}(k)$ represents the hierarchical positional encoding. The physical implication of this design lay in dynamically adjusting the initial search space of the Transformer via Mamba’s outputs, which guaranteed that the generation of residual codebooks at each layer strictly followed the macroscopic motion trends.

Although the input of Mamba contained environmental prefix information, the inherent state compression mechanism of SSM inevitably incurred the loss of high-frequency spatial information. To compensate for this drawback, this work adopted a cross-attention mechanism to achieve visual feature realignment. During decoding, the query vector $Q_{t,k}$ carrying temporal action context globally interacted with the multimodal context $C_{ctx}$ to output task-relevant visual features $R_{t,k}$. To integrate fine-grained visual perception while preserving the macroscopic motion constraints derived from Mamba, residual connections were introduced to inject visual features into action queries:

(16) $$h_{t,k}=\mathrm{LayerNorm}(Q_{t,k}+R_{t,k}),$$

This step realized feature fusion from semantic planning to visual execution. When the model needed to determine tiny displacements at the k-th hierarchy, $Q_{t,k}$ maintained the overall motion trend, while $R_{t,k}$ provided geometric corrections according to object edges or grasp points. This design effectively remedied the shortcomings of state-space models on tasks requiring precise visual alignment.

Finally, the fused feature vector was fed into a linear projection layer $W_{head}\in {\mathbb{R}}^{V\times d}$ (where V denotes the codebook size) to map the feature space onto the logits space. The softmax activation function was subsequently adopted to calculate the probability distribution of discrete codewords at the current hierarchy:

(17) $$l_{t,k}=W_{head}h_{t,k}^{T}+b_{head},$$

(18) $$P\left(z_{t,k}=v|C_{ctx},h_{t}^{T},z_{t,<k}\right)=\frac{\exp\left(l_{t,k}^{\left(v\right)}\right)}{\sum_{j=1}^{V}\exp\left(l_{t,k}^{\left(j\right)}\right)},$$

where $I_{t,k}^{\left(v\right)}$ represents the logit value corresponding to the v-th codebook index, and $b_{head}$ stands for the bias term.

In summary, the generation procedure of the entire hybrid hierarchical architecture was formally formulated as the factorization of a joint probability distribution. For a skill sequence $Z_{t}$ with sequence length H and hierarchical layers K, its generation probability was defined as follows:

(19) $$P\left(Z_{t}|o_{t},l\right)=\prod_{t=1}^{H}\prod_{k=1}^{K}P_{\theta }\left(z_{t,k}|C_{ctx},h_{t}^{T},z_{t,<k}\right),$$

Here, $h_{t}^{T}$ denotes the temporal context encoded by Mamba2, which compresses the full history of previous skill tokens $z_{<t}$ into a linear-complexity hidden state; $C_{ctx}$ is the multimodal perception context from Section 3.4.1; and $z_{t,<k}$ denotes the previously generated residual levels within the same timestep t. The factorization therefore explicitly separates the two dependency dimensions: the temporal dimension is captured once per timestep by $h_{t}^{T}$ through Mamba2, while the hierarchical dimension is modeled autoregressively by the Transformer decoder through $z_{t,<k}$, matching the two-stage generation procedure described above.

## 4. Results and Discussion

To comprehensively verify the effectiveness and simulation-level robustness of HMSL-RFSQ, this work conducted multi-level experiments on the LIBERO and Meta-World benchmarks [21,22]. Through horizontal comparisons with mainstream discretization algorithms and end-to-end methods, this work evaluated the model’s overall performance in multi-task learning and long-sequence inference. Parameter sensitivity experiments and ablation studies were performed to determine the optimal configuration and analyze the individual contribution of each module. Finally, visual analysis was adopted to intuitively demonstrate the superiority of the proposed model in terms of physical manipulation precision and semantic representation capability.

### 4.1. Experimental Setup

#### 4.1.1. Simulation Environments

**Figure 6:**
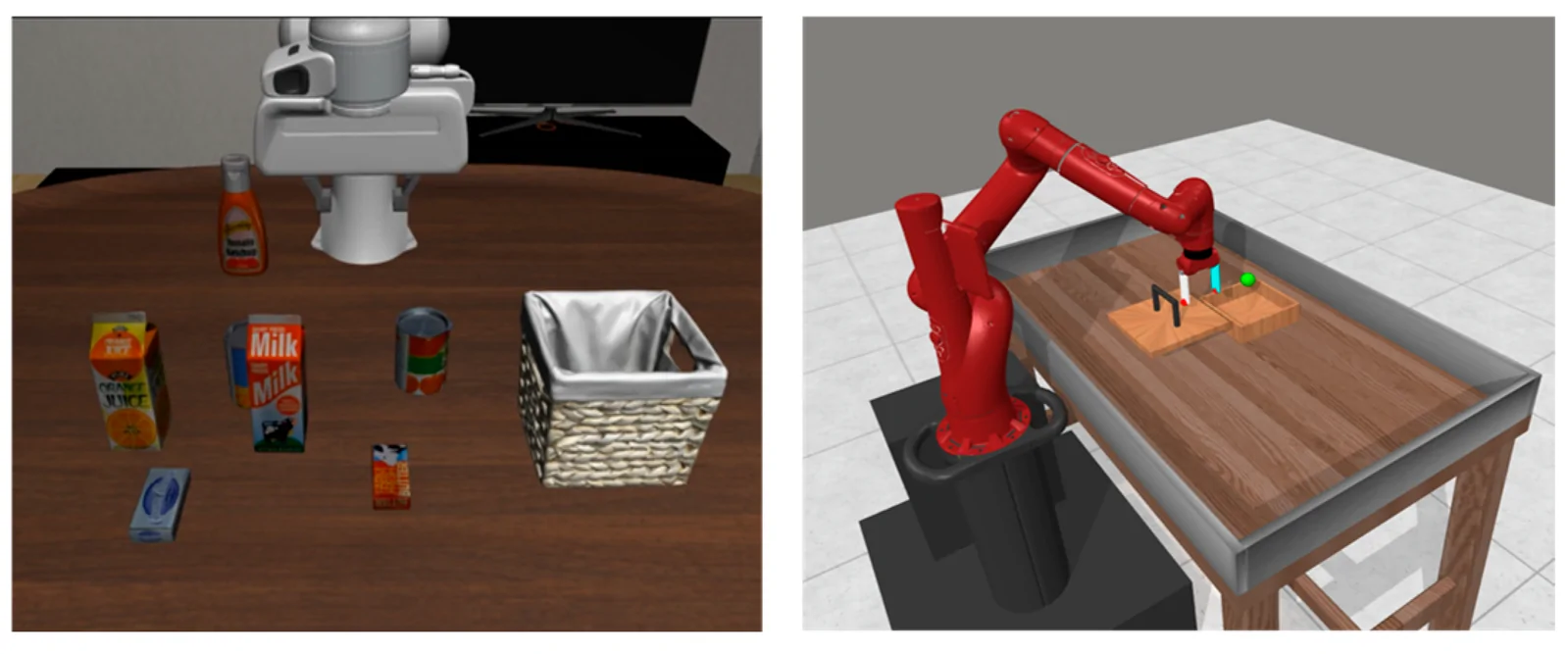
LIBERO (**left**) and Meta-World (**right**) simulation environments.

This work drew on the benchmark environment configurations adopted by QueST and other existing methods [40]. Two high-fidelity physical simulation benchmarks, LIBERO and Meta-World, were selected as experimental platforms, as illustrated in Figure 6. Both platforms are built based on the MuJoCo physics engine and provide realistic rigid-body dynamics simulations alongside abundant human–robot interaction scenarios.

In the experiments of this work, LIBERO was primarily utilized to assess the model’s capabilities in long-horizon task planning and multi-task learning; thus, the two most challenging task subsets within LIBERO were chosen. The first subset, LIBERO-90, contains 90 distinct manipulation tasks covering diverse atomic skills including pick-and-place, object stacking, and articulated object manipulation. The second subset, LIBERO-10, consists of 10 complex long-horizon tasks, each composed of multiple sequentially dependent subgoals. These tasks feature an average temporal span of more than 400 timesteps and strict logical causality constraints.

To verify the cross-domain adaptability of the model across environments with distinct physical dynamics, the Meta-World (ML45) benchmark was introduced. This benchmark includes 45 training tasks (such as window opening and box pushing) and five unseen test tasks. Compared with LIBERO, it exhibits notable discrepancies in physical properties (e.g., friction and gravity), robotic manipulator configuration, and observation viewpoints. To address the absence of textual labels in this benchmark, one-hot encoding of task indices was adopted as an alternative representation. Consequently, the CLIP language encoder and language-conditioned FiLM modulation are not exercised on ML45; the multimodal perception fusion claims are validated on the language-instructed LIBERO benchmarks, whereas ML45 evaluates cross-domain action representation and temporal modeling under task-index conditioning.

#### 4.1.2. Implementation Details and Evaluation Protocol

All models, including the five baselines and HMSL-RFSQ, were implemented in Python 3.10 with PyTorch 2.2.0 and trained on a single NVIDIA RTX 3090 GPU (24 GB). The five baselines were re-trained using their official open-source implementations under the identical demonstration sets and evaluation protocol, adopting their reported default settings with the same tuning budget. Every method was trained on the same 100 expert demonstrations per task, and no method was provided with additional data. HMSL-RFSQ adopts the hyperparameters listed in Table 1. For a fair comparison, each task was evaluated over 50 closed-loop rollouts using a deterministic policy; an episode was counted as successful only when all task-specific subgoals were completed within the maximum episode length, and the reported success rate is the percentage of successful rollouts.

### 4.2. Model Parameter Configurations and Training Objectives

#### 4.2.1. Main Model Hyperparameter Configurations

The specific hyperparameter configurations of the model were extensively verified through experiments to maximize the performance of the model architecture under limited computational resources. The model mainly consisted of two components: an action representation network for action discretization and a skill generation network for sequence modeling. The core parameter settings are summarized in Table 1.

To achieve efficient coverage of the continuous action space under a limited codebook capacity, we set the depth of residual quantization to two layers, and the scalar grid hierarchy of single-stage quantization was elaborately designed as [8, 5, 5, 5]. This parameter configuration yielded 1000 valid discrete index combinations for each residual layer. Specifically, the four scalar levels [8, 5, 5, 5] define the dimensionality of a single FSQ grid and are not distributed across the residual stages; each of the K = 2 residual layers independently applies the same four-dimensional grid, producing 8 × 5 × 5 × 5 = 1000 discrete entries per layer. Compared with conventional binary quantization schemes, such as $2^{N}$, this non-uniform design based on distinct prime bases substantially reduced quantization dead zones and boosted the capability to capture subtle action amplitude variations. The hidden layer dimension of both encoder and decoder was uniformly set to 256. This dimension guaranteed sufficient feature representation capacity while effectively restraining the total number of model parameters, making the architecture suitable for edge deployment on robotic hardware. The model incorporated six Mamba2 blocks to capture temporal dependencies of actions along the time axis. Benefiting from its state-space model with linear computational complexity, Mamba2 efficiently captured evolving action trends spanning multiple timesteps and avoided the explosion of quadratic complexity that plagued Transformer when processing long sequences. The architecture adopted four layers of Transformer decoder to model dependencies across different residual layers K within a single timestep. Since the value of K remained relatively small, the self-attention mechanism of the Transformer accurately modeled strong correlations between coarse-grained actions and fine-grained residual signals, guaranteeing that the generated actions complied with physical structural constraints.

#### 4.2.2. Training Objectives and Loss Functions

In view of the heterogeneous characteristics of HMSL-RFSQ, the training of the entire model was decoupled into two separate optimization stages. The framework first constructed a discrete action representation space and then built the mapping from multimodal perceptions to actions on top of this action space. During the action representation learning stage, the model trained the action representation network in a self-supervised manner. The core objective of this stage was to compress high-dimensional continuous actions into non-uniform fixed scalar grids while maximizing the reconstruction fidelity of actions. The overall loss function $L_{\mathrm{rep}}$ consists of a primary action sequence reconstruction loss and a consistency loss that constrained the distribution of the latent space:

(20) $$L_{\mathrm{rep}}=\lambda_{1}L_{\mathrm{rec}}+\lambda_{2}L_{\mathrm{consist}},$$

(21) $$L_{\mathrm{rec}}=∥a_{t:t+T-1}-{\hat{a}}_{t:t+T-1}{∥}_{1},$$

(22) $$L_{\mathrm{consist}}=\frac{1}{K}\sum_{k=1}^{K}{\left\|r_{k-1}-{\hat{r}}_{k}\right\|}_{2}^{2},$$

(23) $${\hat{r}}_{k}=\frac{{\mathrm{FSQ}}^{-1}\left(\mathrm{FSQ}\left(\alpha_{k}\cdot r_{k-1}\right)\right)}{\alpha_{k}}.$$

The core action sequence reconstruction loss $L_{\mathrm{rec}}$ aimed to minimize the discrepancy between predicted action sequences and raw expert demonstration sequences after the complete encoding–quantization–decoding pipeline. The expert action sequence starting at timestep t with length T is denoted as $a_{t:t+T-1}$, and the sequence reconstructed by the model is marked as ${\hat{a}}_{t:t+T-1}$. Furthermore, an adaptive quantization consistency loss $L_{\mathrm{consist}}$ is introduced to address the “dead code” issue caused by residual magnitude decay as layers deepened in multi-stage residual quantization, where $r_{k-1}$ represents the residual input of the layer k, ${\hat{r}}_{k}$ stands for the reconstructed value obtained after quantization and inverse rescaling, and $a_{k}$ denotes the learnable scaling coefficient of the layer k. $L_{\mathrm{consist}}$ is defined as the mean squared error between the input vector and the reconstructed vector. It not only constrains continuous latent features to gravitate toward discrete quantization centroids but also drives the coefficients $a_{k}$ to dynamically learn optimal amplification factors. This enables the quantizer to adaptively capture subtle residual signals and drastically reduces the cumulative error brought by multi-level quantization.

The policy learning stage focused on learning the mapping from perceptual inputs to action intentions within the frozen discrete latent space. This staged, frozen-codebook design follows the standard VQ-VAE-style training paradigm adopted by VQ-BeT, QueST, and MambaSkill: it stabilizes the discrete representation space and prevents co-adaptation between the codebook and the policy, ensuring that token semantics remain fixed during policy learning. Furthermore, because all ablation variants in Section 4.5 share this identical training schedule, the performance differences among them isolate the effects of the architectural components rather than the training procedure. Since the representation network has achieved low reconstruction error and high reconstruction accuracy via multi-stage residual quantization, the task of this stage was formulated as a standard discrete sequence classification problem. The overall loss function $L_{\mathrm{prior}}$ only comprises the negative log-likelihood loss. Given the environmental observations $o_{t}$ at the current timestep, natural language instructions l, and the corresponding ground-truth discrete index sequence of actions, the training objective was to minimize the discrepancy between the predicted distribution and the ground-truth distribution.

(24) $$L_{\mathrm{prior}}=-\sum_{i=1}^{H}\sum_{k=1}^{K}\log p_{\theta }(z_{i,k}|o_{t},l,z_{<i},z_{i,<k}),$$

By minimizing this loss, the policy network learned to imitate the decision-making patterns of experts within the discrete latent space. Accordingly, during inference, it retrieved skill vectors from environmental inputs and generated reasonable action sequences through the decoder.

### 4.3. Comparative Experiments

To evaluate the overall performance of HMSL-RFSQ including multi-task learning, long-sequence inference and general manipulation capabilities, comparative experiments were conducted on the LIBERO-90, LIBERO-10 and ML45 datasets. This work selected five representative algorithms as comparative baselines, covering mainstream technical routes ranging from end-to-end policies to hierarchical policies and from traditional approaches to state-of-the-art cutting-edge techniques. The compared baselines are detailed below.

1. ACT [8]: A Transformer-based conditional variational autoencoder (CVAE), which generates temporally extended action sequences by decomposing behaviors into action chunks;
2. Diffusion Policy [6]: Which is implemented based on the UNet architecture and maps Gaussian noise to action trajectories through a learned conditional denoising diffusion process;
3. VQ-BeT [34]: A method that combines VQ-VAE for discrete latent space learning with Transformer for latent codebook prediction;
4. QueST [40]: Which constructs discrete latent spaces using FSQ and adopts a causal Transformer for action sequence modeling;
5. MambaSkill [41]: Which constructs discrete latent spaces by combining a Mamba-based autoencoder with FSQ and leverages a two-layer Mamba-augmented autoregressive generator for skill sequence modeling.

Notably, large-scale vision–language–action (VLA) models such as OpenVLA, RT-2, π_0_, and RDT are discussed in Section 2 as the broader research context but are not included in the quantitative comparison. These models rely on large-scale cross-embodiment pretraining with billions of parameters and, in some cases, proprietary or unreleased training corpora, whereas all baselines in Table 2 are trained from scratch under the identical demonstration-only setting. A direct comparison would therefore confound architectural design with data scale and compute budget; we consequently limit our claims to improvements over baselines evaluated under matched training conditions.

Each task was evaluated for 50 runs to calculate success rates in the experiments, and the experimental results are summarized in Table 2. The results showed that on the standard LIBERO-90 dataset, HMSL-RFSQ outperformed the best baseline MambaSkill by 2.9%. Meanwhile, on the ML45 tasks, HMSL-RFSQ tied for second place with the classic ACT, with a mere 0.2% gap behind the top-performing QueST. This demonstrated the outstanding performance of HMSL-RFSQ on basic task learning. It was also noteworthy that on the LIBERO-10 dataset, which consists of complex long-horizon multi-stage manipulation tasks that impose stringent requirements on the model’s temporal modeling capacity, HMSL-RFSQ achieved an excellent success rate of 80.5%. It substantially surpassed MambaSkill, a method renowned for long-sequence modeling, with a performance gain of 3.8%. This strongly demonstrated that HMSL-RFSQ could capture long-horizon temporal dependencies more effectively and mitigate drift during the execution of long-horizon tasks.

Overall, the average success rate of HMSL-RFSQ reached 86.8%, which was remarkably higher than the classic ACT (61.80%) and Diffusion Policy (71.67%), and achieved a 2.3% improvement compared with the best competing baseline. These results fully demonstrated the rationality and superior performance of the manipulation skill learning framework proposed in this work.

Although no dedicated perturbation tests are conducted in this study, the experiments above provide simulation-level evidence of robustness along three axes. First, cross-domain robustness is examined by the two benchmarks, which differ in friction, gravity, manipulator configuration, and observation viewpoints; HMSL-RFSQ remains competitive on both benchmarks (89.8% on LIBERO-90 and 90.1% on ML45, as shown in Table 2). Second, hyperparameter robustness is reflected in the sensitivity sweeps (Section 4.4), where performance degrades gracefully around the selected downsampling factor, codebook size, and residual depth rather than collapsing under small configuration changes. Third, long-horizon robustness is probed by the LIBERO-10 tasks, each spanning more than 400 timesteps with sequentially dependent subgoals, on which HMSL-RFSQ attains an 80.5% success rate. We note that these results do not constitute a formal robustness certification: controlled perturbation tests (observation and action noise, visual distractors) and variance across independent training seeds are not reported and are left as future work (Section 5).

### 4.4. Parameter Sensitivity Analyses

To explore the optimal model configuration for the target tasks, this section focused the analysis on three critical hyperparameters—the temporal downsampling factor, codebook capacity for quantization, and the number of residual FSQ layers—and verified their specific impacts on final task performance. All sensitivity sweeps were conducted on LIBERO-90 to determine the hyperparameter configuration; the selected values were then frozen and applied without retuning to LIBERO-10 and ML45. The competitive performance on these two held-out benchmarks (Table 2) therefore serves as a cross-benchmark validation that the chosen configuration is not overfit to LIBERO-90.

**Figure 7:**
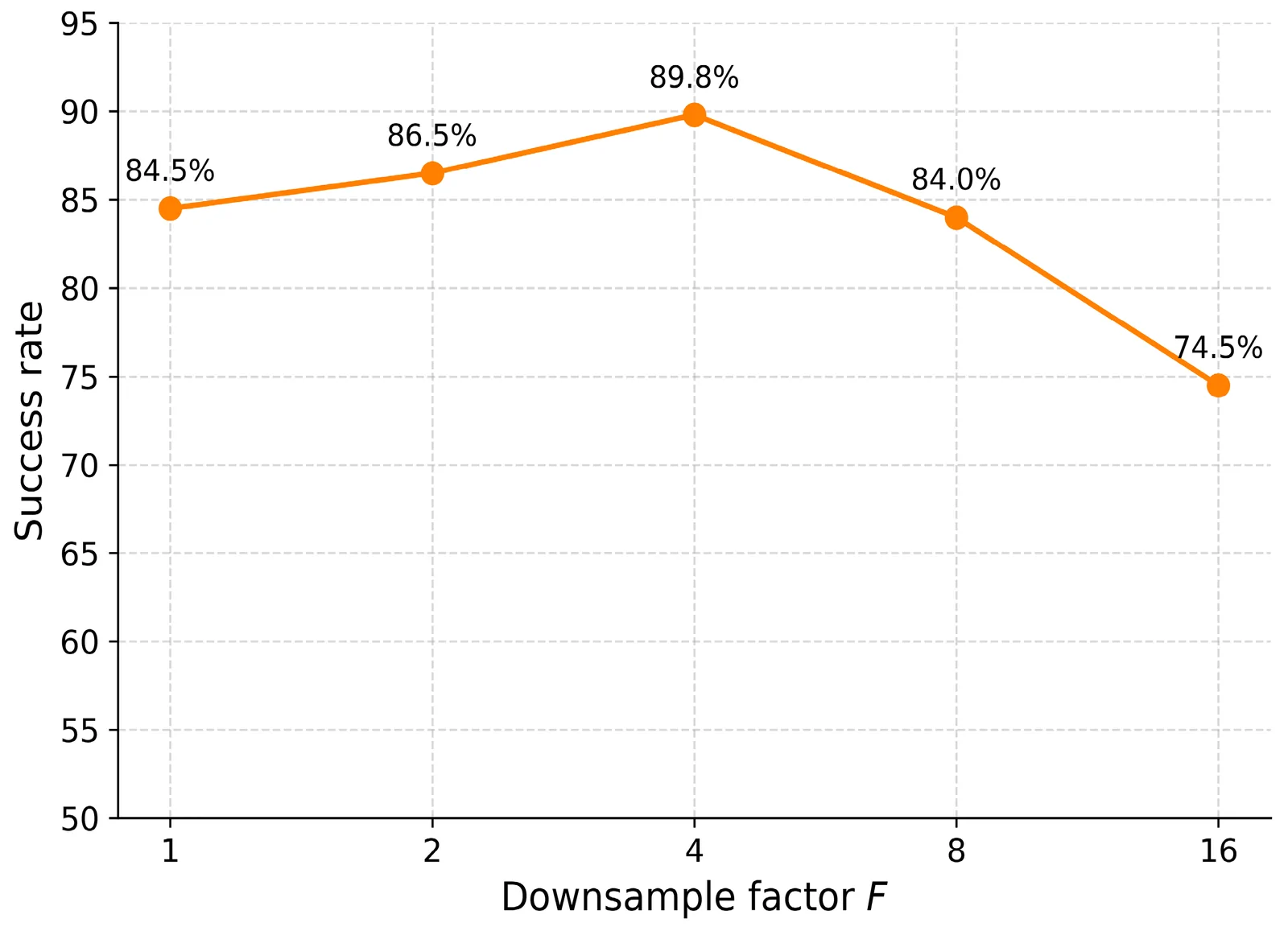
Performance under different downsampling factors.

The temporal downsampling factor governed the compression ratio from raw actions to the latent skill space. To strike an optimal balance between fine-grained motion control and long-horizon temporal rollout, this experiment conducted sensitivity analysis on different temporal downsampling factors to validate their concrete effects on final task success rates. Five distinct configurations $F\in \left\{1,2,4,8,16\right\}$ under fixed-length action chunk T=32 were compared on the LIBERO-90 dataset, and the experimental results are illustrated in Figure 7.

Initially, the task success rate rose as the downsampling factor increased and peaked when the factor equaled 4, followed by a gradual decline. The primary reasons were as follows. An excessively small downsampling factor generated overly long sequences of skill tokens whose lengths approximated the original raw action sequences. The Transformer in the second stage then needed to predict much longer token sequences, which raised the difficulty of sequence modeling and degraded overall performance. By contrast, an excessively large downsampling factor caused each individual skill token to cover an overlong temporal span, resulting in the loss of fine-grained action details and producing overly coarse skills.

**Figure 8:**
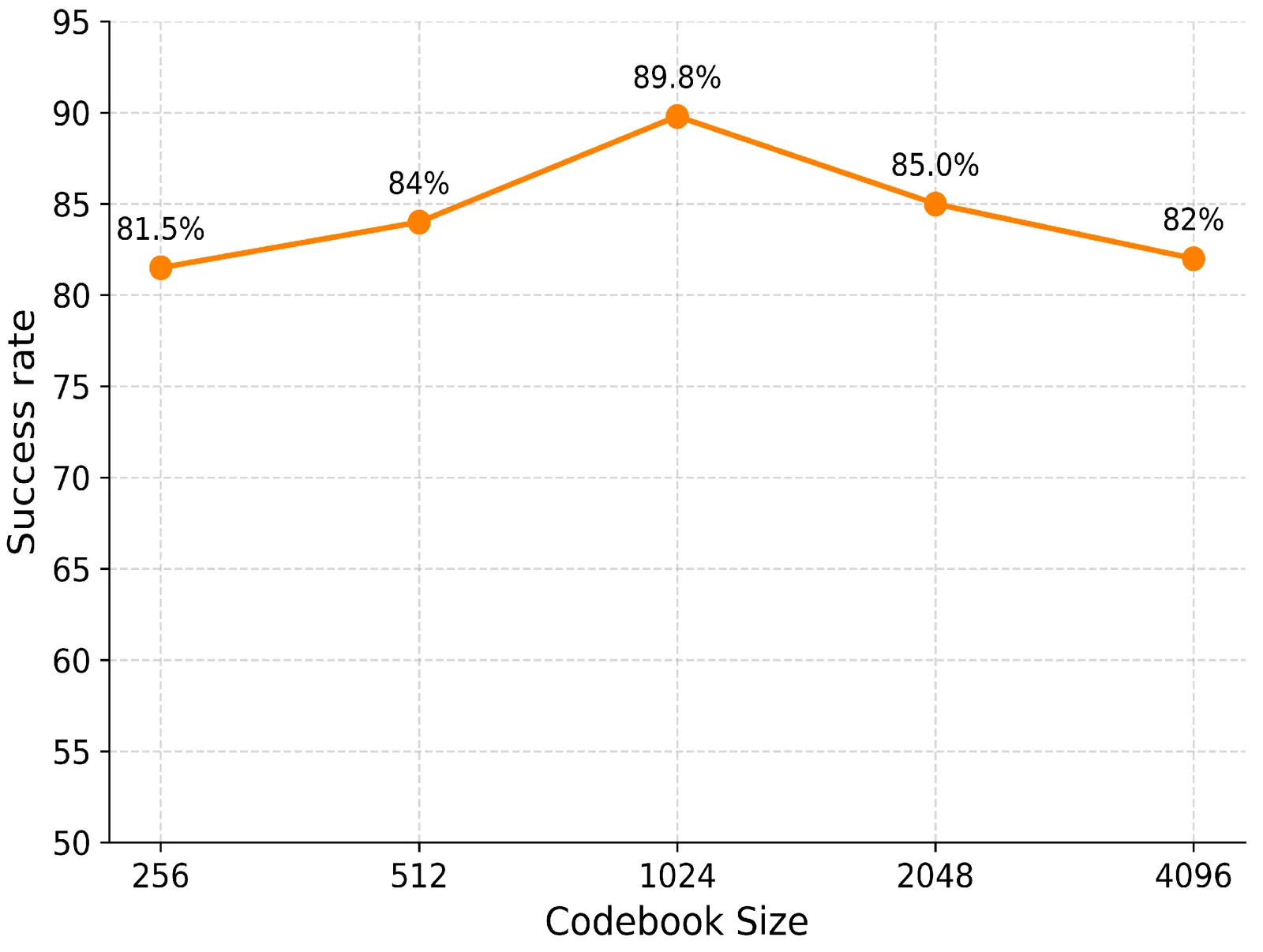
Effect of codebook size on experimental performance.

The codebook capacity of each FSQ layer within RFSQ determined the granularity of discrete actions. An undersized codebook triggered underfitting, including the failure to represent complex actions and massive loss of fine-grained action details; by contrast, an oversized codebook drastically boosted the difficulty of categorical prediction for the skill generation network. This work compared six quantization configurations with codebook sizes set to 128, 256, 512, 1024, 2048 and 4096. The experimental results are shown in Figure 8. The model achieved optimal performance at a codebook size of 1024, which indicated that 1024 served as the optimal codebook size for LIBERO-90 tasks.

**Figure 9:**
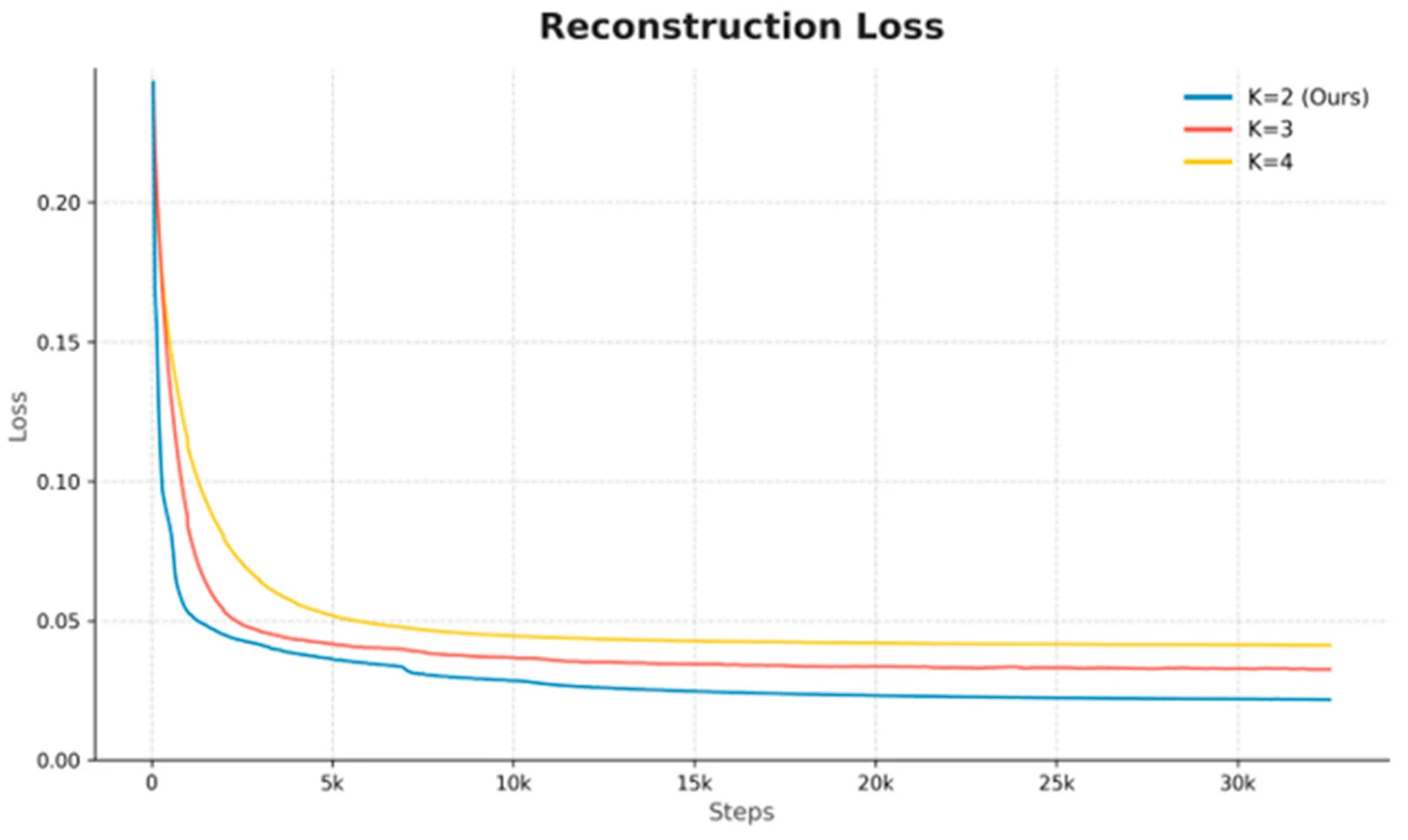
Action reconstruction loss under different residual layer numbers.

**Figure 10:**
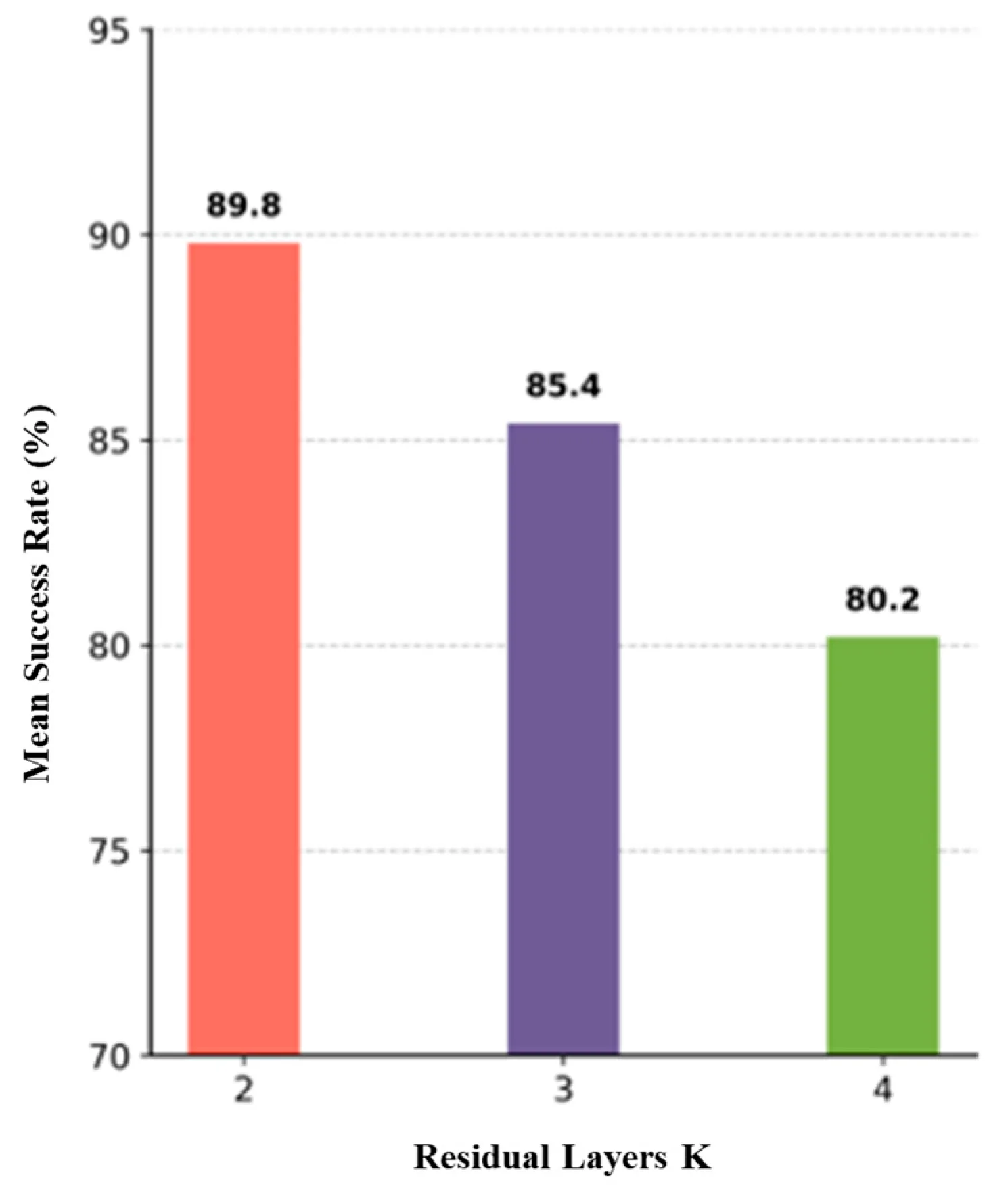
Task success rate for different numbers of residual layers on LIBERO-90.

Theoretically, deeper residual layers enabled discrete representations to reconstruct continuous actions more faithfully. Nevertheless, FSQ was prone to residual magnitude decay, which introduced noise during discretization. Although the RFSQ adopted in this work incorporated an adaptive scaling mechanism for mitigation, residual quantization still introduced high-frequency noise once the number of residual layers exceeded a certain threshold. Meanwhile, extra residual layers imposed heavier prediction burdens on the second-stage skill generation network and degraded model performance. In this experiment, we fixed the single-layer FSQ dimension to 1000 and conducted tests with three residual layer settings: K = 2, K = 3 and K = 4. The experimental results are illustrated in Figure 9 and Figure 10. The experimental curves revealed that as the number of residual layers increased, the loss descent of the trained action representation network became gentler with a higher final converged loss value, accompanied by declining task success rates. Accordingly, the optimal number of residual layers for the current task scenarios was 2.

### 4.5. Ablation Studies

To comprehensively understand the contribution of each key module in the model, this work conducted ablation studies by systematically removing core components to investigate their effects on model performance during the execution of relevant tasks. Specifically, several model variants were adopted to investigate the contributions of different key modules to model performance, as detailed below.

- Remove the Mamba2 module and replace the Mamba-Transformer with a causal Transformer architecture (which processes temporal and hierarchical dependencies simultaneously through causal self-attention) to verify the advantages of the hybrid architecture for modeling long-horizon dependencies.
- Adopt single-layer FSQ for action discretization.
- Eliminate the adaptive scaling mechanism, and employ the original two-layer RFSQ.

The results of ablation studies are listed in Table 3. After removing the Mamba2 module and adopting a Transformer to predict hierarchies autoregressively, the model performance degraded significantly. The task success rate dropped by 5.5% on LIBERO-90 and 7.8% on LIBERO-10. This demonstrated that the Mamba-Transformer adopted in HMSL-RFSQ could effectively capture and model long-horizon dependencies for long-horizon manipulation, enhancing the learning capacity for long-horizon tasks. Moreover, the decline in success rate under single-layer FSQ indicated that discretizing actions via the single-layer FSQ mechanism introduced certain discretization loss, which degraded the overall task execution performance. Meanwhile, the original two-layer RFSQ produced considerable performance degradation of up to 8.6% on LIBERO-90 and 9.5% on LIBERO-10. This revealed that the residual quantization mechanism equipped with the adaptive scaling mechanism realized superior action discretization and incurred less loss of fine-grained action details. This performance drop is consistent with the reconstruction loss curves in Figure 9: without adaptive linear scaling, the second-layer residual decays into the near-zero dead zone of the tanh-based FSQ grid, forcing deeper layers to output near-constant codes and increasing reconstruction error.

### 4.6. Visualization Analyses

**Figure 11:**
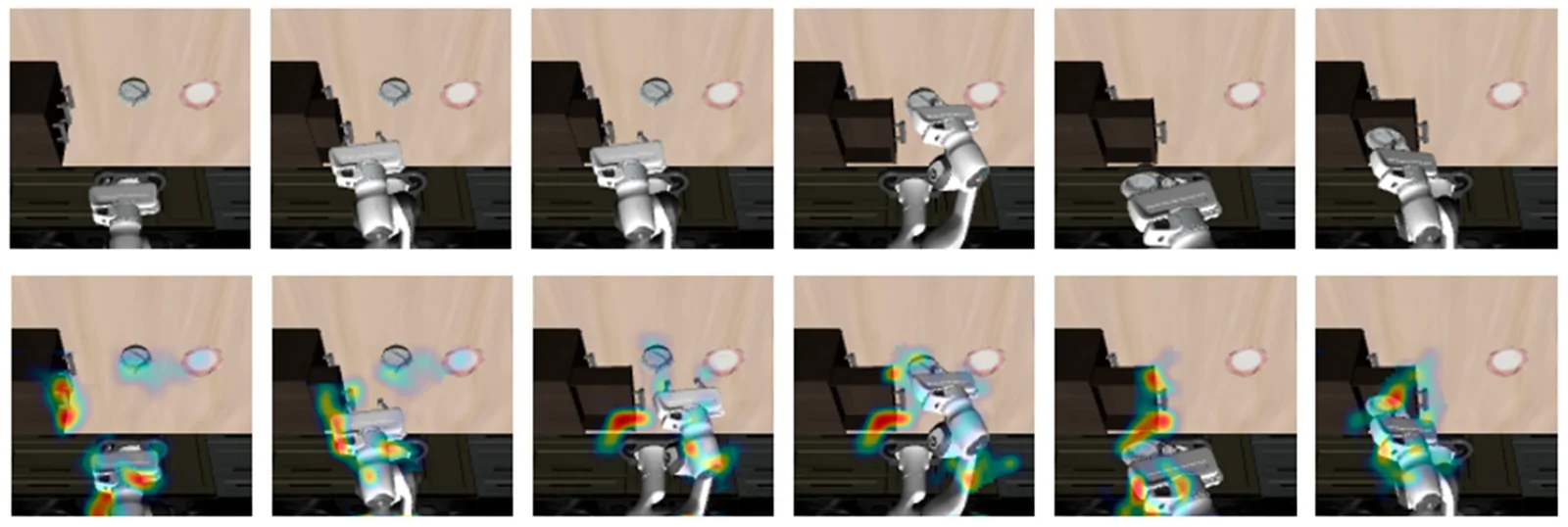
Attention heatmaps during task execution; red areas correspond to high attention weights.

To investigate the feature-focusing mechanism of the policy network when processing high-dimensional visual inputs and its attention toward task-relevant objects during task execution, this work adopted Grad-CAM to generate heatmap visualizations of the final-layer feature maps output by the visual encoder at different timestamps throughout selected tasks, as illustrated in Figure 11. Obvious observations drawn from the results revealed that attention hotspots converged on the end-effector of the robotic manipulator and the contact regions of target objects during task execution. Such semantic-level feature alignment verified that HMSL-RFSQ effectively filtered irrelevant information and enabled the policy network to make valid decisions based on objects and their interactive relationships implied within instructions.

**Figure 12:**
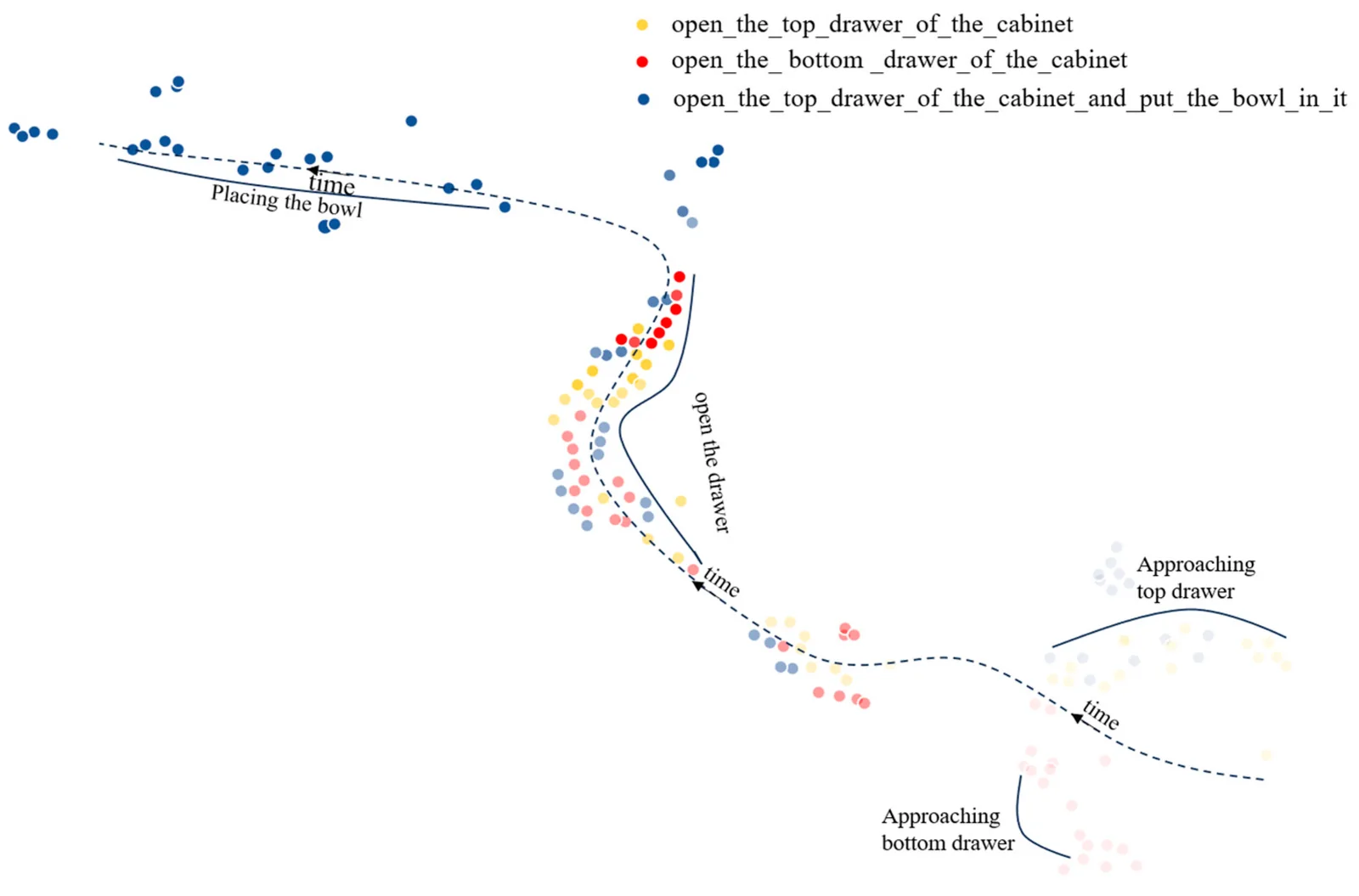
The visualization results of t-SNE.

To determine whether the action representation network learned discriminative abstract skill representations in the latent space, this experiment examined the corresponding embedding vector Z for combinations of several similar tasks. Feature vectors from different task stages were extracted during the experiment, and the t-SNE (t-distributed stochastic neighbor embedding) algorithm was employed to reduce their dimensionality to a two-dimensional plane for visualization. The visualization results are shown in Figure 12.

Each dot in the figure denoted the embedding vector Z at a task execution stage. Reduced transparency and deepening color indicated the temporal progression of tasks. It was observed from the figure that different manipulation skills (e.g., approaching, open, placing) formed compact, well-defined clusters in specific spatial regions. This demonstrated that the model could learn fine-grained, structured skill-action representations. Such representations provided a reliable semantic foundation for high-level task planning, enabling the model to accurately retrieve and execute corresponding skill actions according to information such as the current state and task instructions.

**Figure 13:**
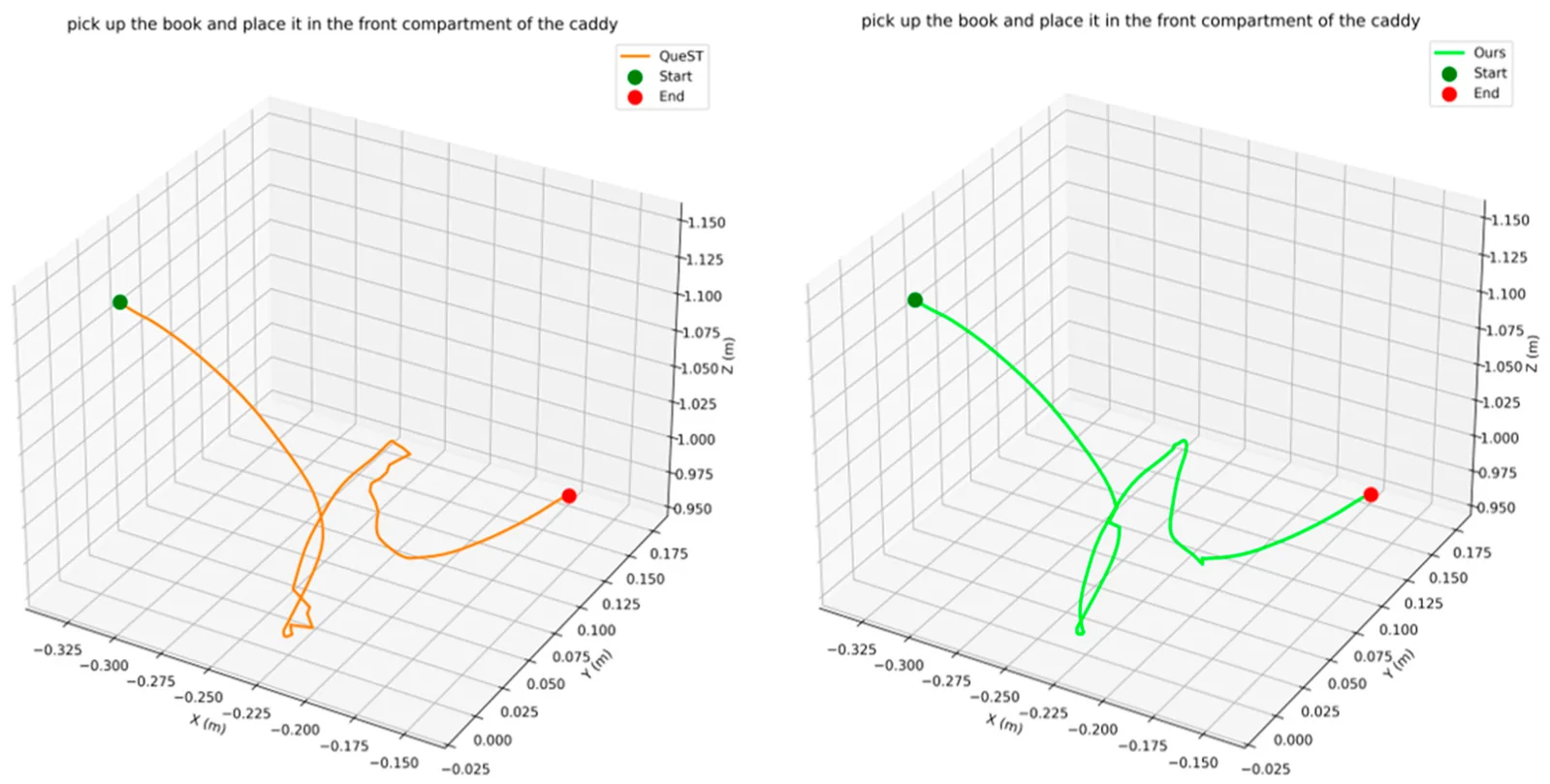
Visual comparison of trajectories (QueST on the left, HMSL-RFSQ on the right).

In addition, this work performed a visual comparison of end-effector motion trajectories between HMSL-RFSQ and QueST on selected representative tasks, and the results are illustrated in Figure 13. It could be observed from the trajectory plots that the trajectories produced by the QueST model contained considerable jitter and noise and exhibited poor smoothness. By contrast, the trajectories generated by HMSL-RFSQ maintained high smoothness and continuity with only minor fluctuations. This indicated that the action representation network proposed in this work well preserved action details, sustained favorable action coherence during decoding, and effectively alleviated the high-frequency jitter problem commonly seen in discretized policies. It also demonstrated that skill generation network designed in this work predicted skill tokens with relatively high precision.

## 5. Conclusions

This work investigated an approach for manipulation skill learning based on discrete generative policies. It aimed to address execution challenges for robotic manipulators in complex task scenarios and enhance the operational efficiency and precision of robots in multi-stage tasks. This work proposed a hierarchical manipulation skill learning framework based on RFSQ. A robust RFSQ equipped with an adaptive scaling mechanism was introduced, which mitigated the residual decay problem via learnable scaling factors. It realized compact discrete representations while preserving original action details to the greatest extent. On this basis, a hybrid skill generation architecture integrating Mamba2 and Transformer was constructed. It leveraged Mamba’s superior memory capability for long-sequence modeling to alleviate long-context information degradation and combined the Transformer with it to precisely capture complex dependencies across skill hierarchies. A series of experiments demonstrated that the proposed method substantially enhanced the execution coherence and success rate of robots in long-horizon manipulation tasks.

However, the skill learning policy of this work mainly relies on conditional imitation learning. Although the hybrid architecture of Mamba2 and Transformer realizes outstanding performance in long-sequence modeling for manipulation, it still lacks sufficient adaptive adjustment capability when confronted with environmental disturbances. Future work can explore introducing online reinforcement learning or human-in-the-loop correction mechanisms based on the pre-trained model. Robots collect feedback data through real-time interaction with the environment to continuously fine-tune the weights of the policy network, which endows the model with self-correction and dynamic adaptation capabilities during the execution of long-horizon tasks. In addition, all reported quantitative results are from a single training seed; evaluating statistical significance over multiple independent training runs remains important future work.

Furthermore, all experiments in this study are conducted in MuJoCo-based simulators (LIBERO and Meta-World); no real-robot deployment, domain randomization, or sim-to-real transfer analysis has been performed. Although the two benchmarks differ in friction, gravity, manipulator configuration, and observation viewpoints, and thus provide preliminary evidence of robustness across distinct simulated physics settings, they cannot fully reproduce sensor noise, actuator dynamics, and real contact friction. In particular, the adaptive scaling factor $\alpha_{k}$ is learned from simulated residual distributions and may require recalibration on physical hardware. In future work, we plan to (1) introduce domain randomization over action noise, friction coefficients, and actuator latency during action representation training and (2) conduct a pilot real-robot study to validate the reconstruction fidelity and closed-loop performance of RFSQ under physical conditions.

Finally, several controlled studies remain as future work to more rigorously isolate the source of the gains: (1) a continuous action head on the same hybrid Mamba2–Transformer backbone to separate the contribution of RFSQ discretization from that of the architecture; (2) a controlled removal of the FiLM/CLIP multimodal fusion module; (3) a direct comparison between the two-stage frozen-codebook training and joint end-to-end optimization; and (4) a factorial interaction analysis between residual depth K and temporal modeling capacity, complementing the present single-factor sweeps. Moreover, no dedicated long-context retention or memory-ablation experiment is conducted, so the evidence for improved temporal modeling remains indirect (the LIBERO-10 success rate and the Mamba2-removal ablation in Table 3); a controlled memory-retention benchmark will be pursued together with the multi-seed evaluation noted above.

## Figures and Tables

**Table 1:** Main hyperparameter configurations of the model.

Modules	Hyperparameters	Values
Action Representation Network	Encoder Dimension	256
	Decoder Dimension	256
	Encoder Layers	4
	Decoder Layers	4
	FSQ Levels per Residual Layer	[8, 5, 5, 5]
	Conv Kernel/Stride	[5, 3, 3]/[2, 2, 1]
	Action Chunk Size	32
Skill Generation Network	Model Dimension	512
	Mamba Layers	6
	Mamba State Dimension	32
	Transformer Layers	4
	Transformer Heads	4
	Vocab Size	1000

**Table 2:** Results of comparative experiments. Note: All results are from a single training run; each task is evaluated over 50 episodes. Standard deviations over multiple independent training seeds are not reported.

Methods	LIBERO-90	LIBERO-10	ML45	Average
ACT	50.8%	44.5%	90.1%	61.8%
Diffusion Policy	75.4%	50.1%	89.5%	71.7%
VQ-BeT	79.3%	59.2%	87.2%	75.2%
QueST	86.7%	68.3%	90.3%	81.8%
Mambaskill	86.9%	76.7%	89.9%	84.5%
HMSL-RFSQ	89.8%	80.5%	90.1%	86.8%

**Table 3:** Results of ablation studies.

Methods	LIBERO-90	LIBERO-10
HMSL-RFSQ	89.8%	80.5%
Without Mamba2 module	84.3%	72.7%
Single-layer FSQ	85.3%	75.8%
Original two-layer RFSQ	81.2%	71.0%

## Data Availability

The data supporting the findings of this study are available from the corresponding author upon reasonable request.
